# Simultaneous quantification of terpenes and cannabinoids by reversed-phase LC-APCI-MS/MS in *Cannabis sativa* L. samples combined with a subsequent chemometric analysis

**DOI:** 10.1007/s00216-024-05349-y

**Published:** 2024-05-25

**Authors:** Justine Raeber, Michael Poetzsch, Anina Schmidli, Sina Favrod, Christian Steuer

**Affiliations:** 1 Institute of Pharmaceutical Sciences, ETH Zurich, Vladimir-Prelog-Weg 1-5/10, CH-8093 Zurich, Switzerland; 2 Swiss Drug Testing GmbH, Technoparkstrasse 2, CH-8406 Winterthur, Switzerland

**Keywords:** HPLC, Terpenes, APCI, Cannabinoids, Mass spectrometry

## Abstract

*Cannabis sativa* L*.* has been the most discussed medicinal plant in recent years. In particular, the dynamic shift from a formerly illicit and tightly controlled substance to a plant recognized for both medicinal and recreational purposes has brought *C. sativa* into the global spotlight. Due to the ongoing international legalization processes, fast and convenient analytical methods for the quality control of *C. sativa* flowers for medicinal and recreational purposes are of tremendous interest. In this study, we report the development and validation of a liquid chromatography-tandem mass spectrometry (LC–MS/MS)-based method applying atmospheric pressure chemical ionization (APCI) to fully quantify 16 terpenes and 7 cannabinoids including their acidic forms by a single chromatographic method. The method presented here is unique and simple, as it eliminates the need for derivatization reactions and includes the unconventional analysis of volatile compounds by liquid chromatography. Samples were prepared by a simple and fast ethanolic extraction. Separation was accomplished within 25 min on a reversed-phase C18 column. Method validation was conducted according to international guidelines regarding selectivity, accuracy, precision, robustness, and linearity. Detection was done in multiple reaction monitoring, which allowed the simultaneous quantification of co-eluting analytes applying two selective mass transitions. In addition, due to reproducible in-source decarboxylation, the acidic forms of cannabinoids were reliably quantified using mass transitions of the neutral forms. The accuracy given as the bias was below 15% for all analytes. Matrix effects for cannabinoids were studied by spiking *Humulus lupulus* extracts with the analytes at varying concentrations. APCI did not show susceptibility toward ion suppression or enhancement. In addition, the recovery effect after spiking was between 80 and 120% for terpenes. Further, 55 authentic *C. sativa* extracts were fully quantified, and the obtained results for the terpene profiles were compared to state-of-the-art gas chromatography coupled to flame ionization detection. Comparable results were achieved, emphasizing the method’s applicability for cannabinoids and terpenes. Further, acquired metabolite patterns for *C. sativa* samples were studied, identifying a relationship between cannabinoid and terpene patterns, as well as the abundance of myrcene in CBD-dominant *C. sativa* strains.

**Graphical Abstract:**

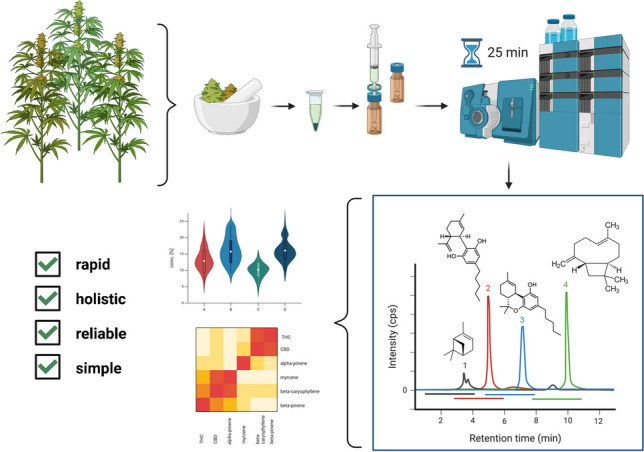

**Supplementary Information:**

The online version contains supplementary material available at 10.1007/s00216-024-05349-y.

## Introduction

In the past century, no other plant has experienced such a dramatic change in public perception as *Cannabis sativa* L. It has transitioned from being an illicit controlled substance to a promising drug candidate for a wide range of medical problems, such as spasticity in multiple sclerosis, neuropathic pain, anorexia, epilepsy, and chemotherapy-induced nausea [1–4]. Although the recreational use of cannabis with a high THC content is still banned in many countries, the legal framework for its medical use is experiencing a dynamic transformation [4–6]. Countries like Switzerland have introduced simplified prescription and dispensing regulations of medicinal cannabis by doctors and pharmacies. As a result, they no longer require an exemption permit from the Federal Office of Public Health (BAG) [7]. As the demand for medicinal cannabis rises, so does the need for quality assurance standards. In response, the European Directorate for the Quality of Medicines and Health Care (EDQM) has initiated the addition of a new monograph for cannabis flowers in the European Pharmacopeia 11.5 (Ph. Eur.) [8]. Although the exact nomenclature and classification of cannabis is still a matter of debate and distinctions between the species and subspecies *Cannabis sativa* L., *ruderalis*, and *indica* have been made and rejected, it is undisputed that cannabis is exceptionally chemical diverse, and classification should be based on its chemical composition [9–11]. The most widely accepted classification method, which has also been incorporated into the cannabis flower monograph of the Ph. Eur. 11.5, is based on subdividing cannabis into different chemotypes based on their tetrahydrocannabinol (THC) and cannabidiol (CBD) content [8]. Although a distinction is made between THC dominant, THC and CBD balanced, and CBD dominant variants, other constituents such as cannabigerol (CBG) and minor cannabinoids are gaining interest [12–14]. Both in vitro and in vivo studies have implied differences in the activity of isolated plant compounds and the whole extract. These observations have given rise to the “entourage effect,” a synergistic interplay among phytochemicals, which manifests itself in enhancing and modulating effects through combinations of phytochemicals [13, 15–17]. With the discovery of the entourage effect, the primary focus on cannabinoids has shifted towards other constituents, such as terpenes. To date, terpenes in cannabis have been mainly valued for their flavor and fragrance profile, but they have their own pharmacological effects. They can interact synergistically with each other and/or with cannabinoids [14, 18]. Methods to analyze the full composition of metabolites in cannabis have gained momentum. Metabolomics is being used as a tool to capture the full chemical diversity of cannabis and refine its classification into chemotypes [6, 19–21]. As a result, it is also becoming increasingly evident that commercial labels on cannabis products, which make claims about a pharmacological effect and a strain, do not reliably match the chemical composition of the chemovar [20]. The growing interest in the composition of cannabis flowers has also promoted the development of analytical methods. Gas chromatography (GC) hyphenated to flame ionization detection (FID) or mass spectrometry (MS) has been preferred for the analysis of terpenes in general. In contrast, liquid chromatography (LC) coupled with an ultraviolet (UV) detector or MS has been favored for cannabinoid analysis [16, 22–24]. GC is well suited for analyzing terpenes, as the analytes are both thermostable and volatile. Moreover, GC provides high separation efficiency [9, 14]. Since terpenes consist of the same repeating isoprene building blocks, they differ only in the number of units and stereochemistry, making separation and mass spectra interpretation challenging [11, 25, 26]. Although GC offers several advantages for terpene analysis, it encounters difficulties in analyzing cannabinoids. The acid forms of cannabinoids undergo partial or complete heat-induced decarboxylation into their neutral form during injection into the GC. To avoid this reaction, various derivatization strategies have been introduced to protect the carboxy groups and improve the volatility of the analytes. However, these derivatization reactions are time-consuming, and achieving complete derivatization is not always possible. For this reason, LC has become the preferred method for cannabinoid analysis [23, 25]. With the currently growing need for reliable characterized and quantified cannabis products, fast, convenient, and greener analytical methods are crucial. To date, no analytical method combines the analysis of cannabinoids and terpenes in a single step, removing the need for time-consuming derivatization reactions. Efforts have been made to analyze terpenes by LC–MS and atmospheric pressure chemical ionization (APCI). However, the study by Hyland et al. was restricted to seven terpenes and did not include cannabinoids [27]. In this study, we developed and validated an LC-APCI-MS/MS method for simultaneously quantifying16 terpenes and 7 cannabinoids. The presented method does not require time and reactant-consuming derivatization and exhibits a shorter run time than most GC methods, leading to a fast and holistic analysis of the major components of cannabis flowers. The presented method takes the complex composition of cannabis flowers into account and allows a simplified chemovar determination. Well-characterized cannabis flowers allow for classification, which in return can be used for clinical research as a standardized and fully characterized phytopharmaceutical. Consequently, this would allow for an in depth understanding of deviating pharmacological properties between products.

## Materials and methods

### Chemicals and reagents

Sabinene (98%) was purchased from abcr (Karlsruhe, Germany); citronellol (95%, Acros Organics™) from Thermo Fisher Scientific (Reinach, Switzerland); ammonium acetate (> 99%) and methanol (Optima™, LC–MS grade) from Fisher Chemicals (Loughborough, UK); menthone (purum), limonene (purum), and propylparaben (purum) from Fluka (Buchs, Switzerland); and β-caryophyllene from Frey + Lau (Henstedt-Ulzburg, Germany), THCA (1 mg/mL in isopropanol), CBD (1 mg/mL in methanol), CBDA (1 mg/mL in acetonitrile), CBDV (1 mg/mL in ethanol), CBG (1 mg/mL in ethanol), Δ^8^-THC (1 mg/mL in methanol), and Δ^9^-THC-D3 (0.1 mg/mL in ethanol) from Lipomed (Arlesheim, Switzerland). Ethanol (Emsure®, ISO, Reag. Ph. Eur., absolute for analysis), carvacrol (SAFC®, ≥ 98%), isobornyl acetate (SAFC®, ≥ 90), myrcene (Sigma, analytical standard), linalool (Sigma, 97%), geranyl acetate (Sigma, analytical standard), THC (Supelco, Cerilliant®, 1 mg/mL in methanol), and CBN (Supelco, Cerilliant®, 1 mg/mL in methanol) were obtained from Merck (Darmstadt, Germany). Citral (> 98%) and α-humulene (> 93%) were bought at TCI (Eschborn, Germany), and formic acid (99–100%, Ph. Eur.) from VWR (Dietikon, Switzerland) and water were obtained from an in-house water purifying system (ELGA Labwater, PURELAB flex 3, 18.2 MΩ).

### LC-APCI-MS/MS method development

The LC–MS/MS system used in this study was a Sciex ExionLC HPLC system coupled to a TripleQuad 3500 MS equipped with an atmospheric pressure chemical ionization (APCI) source (AB Sciex, Redwood City, CA, USA). Chromatographic separation was achieved with a Symmetry® C18 column (4.6 × 100 mm, particle size 3.5 μm) from Waters (Milford, MA, USA) held at 45 °C. A pre-column was installed to prevent premature column degradation (UHPLC polar C18, 2.1 mm ID, SecurityGuard™, ULTRA Cartridges from Phenomenex). Mobile phases A and B consisted of 2 mM ammonium acetate with 0.1% (v/v) formic acid in water and 2 mM ammonium acetate with 0.1% (v/v) formic acid and 5% (v/v) water in methanol, respectively. The injection volume was set to 5 µL. The solvent gradient was as follows: starting conditions were set to 70% B from 0 to 1 min with a flow rate of 0.7 mL/min. The flow was then decreased to 0.6 mL/min, and the gradient was slowly increased to 98% B until minute 20. Conditions were held constant until minute 26.5. Thereafter, the gradient was rapidly decreased to 70% B and held until minute 28. The autosampler was cooled to 15 °C. MS data was acquired in positive mode with the following source conditions: curtain gas (CUR) 20.0 psi, collision gas (CAD) 9 psi, nebulizer current (NC) 5.0 μA, temperature (TEM) 500.0 °C, and ion source gas 1 (GS1) 45.0 psi. Entrance potential (EP) was set to 10.0 V. The declustering potential (DP), collision energy (CE), and collision cell exit potential (CXP) were adjusted individually for each analyte, as specified in Table 1. The MRM was conducted as a scheduled experiment with a detection window of 200 s; the target cycle time across single MRM experiments was set to 1 s, which led to a total of 1680 cycles. Data acquisition was performed using Analyst® (AB Sciex, version 1.7.2.), and processing was carried out using Sciex OS (AB Sciex, version 2.0.0.45330).

**Table 1:** Optimized MRM parameters for terpenes and cannabinoids analyzed with APCI LC–MS/MS. Upper values are for the quantifier and lower for the qualifier

#	Analyte	Retention time [min]	Q1 [Da]	Q3 [Da]	DP [V]	CE [V]	CXP [V]
1	cis-citral	4.18	153.1	41.2	41	35	10
			153.1	69.1	41	17	12
2	carvacrol	4.46	151.1	91.1	51	33	8
			151.1	77.1	51	39	8
3	trans-citral	4.51	153.1	41.2	41	35	10
			153.1	69.1	41	17	12
4	trans-menthone	4.88	155.1	81.1	51	17	8
			155.1	79.1	51	35	6
5	linalool	5.17	137.0	81.1	61	19	8
			137.0	77.1	61	31	6
6	cis-menthone	5.74	155.1	81.1	51	17	8
			155.1	79.1	51	35	6
7	citronellol	6.17	157.0	83.1	36	15	8
			157.0	55.1	36	29	6
8	CBDV	7.77	287.1	165.2	61	31	12
			287.1	123.1	61	51	12
9	isobornyl acetate	8.58	137.0	81.0	61	17	8
			137.0	77.0	61	31	8
10	geranyl acetate	9.06	137.0	81.0	61	17	8
			137.0	77.0	61	31	8
11	CBG	11.18	317.2	193.3	41	31	16
			317.2	123.2	41	41	4
12	CBD	11.20	315.2	193.2	41	33	14
			315.2	123.1	41	45	12
13	CBDA	11.87	315.2	193.2	41	33	14
			315.2	123.1	41	45	12
14	sabinene	13.11	137.1	81.1	26	17	8
			137.1	77.0	26	33	8
15	myrcene	13.45	137.0	81.1	56	19	8
			137.0	77.0	56	31	8
16	CBN	14.09	311.1	178.3	41	83	14
			311.1	152.1	41	103	12
17	β-pinene	14.42	137.1	81.1	26	19	8
			137.1	77.1	26	33	6
18	limonene	15.12	137.1	81.1	26	17	8
			137.1	77.0	26	33	8
19	Δ^9^-THC	15.30	315.1	193.0	41	27	4
			315.1	76.9	41	79	12
20	α-pinene	15.46	137.1	81.1	26	19	8
			137.1	77.1	26	33	6
21	THCA	19.22	315.1	193.0	41	27	4
			315.1	76.9	41	79	12
22	α-humulene	21.13	205.1	93.2	21	31	6
			205.1	109.2	21	23	4
23	β-caryophyllene	21.44	205.1	93.2	21	31	6
			205.1	109.2	21	23	4
**IS**	propylparaben	2.93	181.0	139.1	31	17	10
			181.0	95.1	31	27	10
**IS**	Δ^9^-THC-D3	15.30	318.1	196.0	41	27	4
			318.1	76.9	41	79	12

### Method validation

Method validation was conducted on the basis of the International Council for Harmonization of Technical Requirements for Registration of Pharmaceuticals for Human Use (ICH) Q2(R2) guidelines in regard to accuracy, precision, specificity, linearity, range, and robustness [28]. Accuracy and precision were determined by preparing six calibration standards (Cals) and three quality control (QC) samples in the low, medium and high concentration range. The limit of quantification (LoQ) was placed at the lowest calibrator. Over the course of a week, the Cals and QCs were measured on three separate days. The accuracy is expressed as the bias. The concentrations of the duplicate QC samples were back-calculated using the calibration and the corresponding fitted regression function. The relative difference was calculated between the mean experimental and theoretical concentrations of the QC samples. The precision was investigated as the relative standard deviation and reported as precision for both intra- and inter-day performance in accordance to Peters et al. [29]. The method’s specificity was ensured by the presence of both quantifier and qualifier ions and by directly comparing the ion ratios of both MRM transitions for each analyte. In addition, the relative retention time of an analyte was not allowed to further deviate than + / − 0.02 from the internal standard. To rule out matrix effects, three *Humulus lupulus* samples were spiked at three different concentration levels along the high, medium, and low calibration range to provide an analyte-free matrix for the cannabinoids. Besides being free of cannabinoids, *H. lupulus* contains a terpene composition similar to that of *C. sativa*. These samples were analyzed in triplicate, and the recovery effect (RE) was determined according to Formula (1) [24].

1 $$RE \left[\%\right]=\left(\frac{{conc}_{spiked sample}-{conc}_{authentic sample}}{{conc}_{spiking}}\right)\times 100$$

### Preparation of Cal and QC standards

*C. sativa*, depending on its chemotype, can be categorized into high THC, balanced THC:CBD, or low THC content varieties. While research and legislation have focused primarily on these two psychoactive compounds, minor compounds are gaining significance. To address this growing interest, two calibration and QC mixtures were prepared to cover a wide range of concentrations. To calibrate for minor compounds, two stock solutions with concentrations of 1000 μg/mL for terpenes and 40 μg/mL for cannabinoids were prepared, respectively. Direct dilutions were created by combining these stock solutions in varying proportions. On each validation day, 100 μL of the respective Cal or QC was combined with 20 μL of an IS stock solution, resulting in a final IS concentration of 20 μg/mL for propylparaben and 0.18 μg/mL for Δ^9^-THC-D3. Detailed concentrations for Cal and QC samples of minor components are presented in Table 2. For the detection of major compounds, individual stock solutions for Cals and QCs were prepared by direct dilution of each commercial 1000 μg/mL cannabinoid solution. On the corresponding validation day, each Cal and QC stock solution was further diluted 1:1 with an IS stock solution, resulting in a final IS concentration of 0.18 μg/mL for Δ^9^-THC-D3. The final QC and Cal concentrations can be viewed in Table 3.

**Table 2:** Concentration of Cals and QCs for the analysis of the minor calibration range of *C. sativa*

	Cal 1	Cal 2	Cal 3	Cal 4	Cal 5	Cal 6	QC_High_	QC_med_	QC_low_
Terpene conc. [µg/mL]	250	200	100	50	10	5	225	75	30
Cannabinoids conc. [µg/mL]	20	15	8	5	1	0,1	18	6	0,3
Cannabinoid acids conc. [µg/mL]	20	15	8	5	1	0,5	18	6	2

**Table 3:** Concentration of Cals and QCs for the analysis of the major calibration range of *C. sativa*

	Cal 1	Cal 2	Cal 3	Cal 4	Cal 5	Cal 6	QC_High_	QC_med_	QC_low_
Cannabinoids conc. [µg/mL]	60	40	20	5	1	0.1	55	30	0.25
CBDA [µg/mL]	60	40	20	8	5	1	55	30	3

### Sample extraction

Extraction of authentic *C. sativa* flower samples and processed material was conducted by the Swiss Drug Testing Lab (Winterthur, Switzerland). For each sample, dried *C. sativa* flowers were ground and dissolved in EtOH to reach a final concentration of 20 mg/mL. The samples underwent a 5-min sonication, followed by centrifugation at 4000 RPM for an additional 15 min. In the final step, extracts were filtered using a 0.45-μm PTFE syringe filter. Filtered samples were stored in a freezer at − 18 °C until analysis. Plants were cultivated in Switzerland. Characteristics of samples can be found in the supplementary Table 1.

### Analysis of authentic *C. sativa* samples

Authentic samples were analyzed at two different dilutions in order to cover both major and minor compounds. For minor compounds, the crude ethanolic extract was combined with an IS stock solution, resulting in a final concentration of 20 μg/mL for propylparaben and 0.18 μg/mL for Δ^9^-THC-D3. In the case of major compounds, the crude extract was initially diluted 50-fold with EtOH and then mixed in a 1:1 ratio with an IS stock solution to achieve a final concentration of 0.18 μg/mL Δ^9^-THC-D3. Between every five samples, a blank of EtOH, and after every 10 measurements, a system suitability test consisting of 200 μg/mL α-pinene, β-pinene, α-humulene, β-caryophyllene, and cis- and trans-citral was injected. Further, QC samples were analyzed after every 20 samples.

### Comparison to state-of-the-art methods

In order to compare the efficiency of the LC–MS/MS quantification with that of conventional methods, 15 authentic cannabis samples were additionally analyzed and quantified by a fully validated GC-FID method published by our group previously. A GC Trace 1600 configured with an AI 3000 autosampler (Thermo Fisher Scientific, Waltham, MA, USA) was used for analysis and equipped with a BGB-wax column (30 m × 0.25 mm × 0.25 μm, BGB Analytik, Boeckten, Switzerland). Minor adjustments were made by setting the split ratio to 1:10 [24]. As a carrier gas helium 6.0 (PanGas, Dagmersellen, Switzerland) was used. Data acquisition was performed using Chromeleon (Thermo Fisher Scientific, version 7.3.1.). The same minor calibration solutions were used in order to fully quantify authentic samples using GC-FID.

### Data visualization and analysis

Chromatograms were generated using Python (version 3.11.6), which included the matplotlib and seaborn libraries. Pearson correlation and violin plots were created using GraphPad Prism (version: 10.1.2). Principal component analysis (PCA) was conducted using MATLAB (version: Release R2022b, version: 9.13.0; The MathWorks Inc.). Prior to analysis, the data underwent pre-processing by excluding variables with zero entries and auto-scaling. Graphical abstract was created with biorender.com.

## Results and discussion

### Method development

**Fig. 1:**
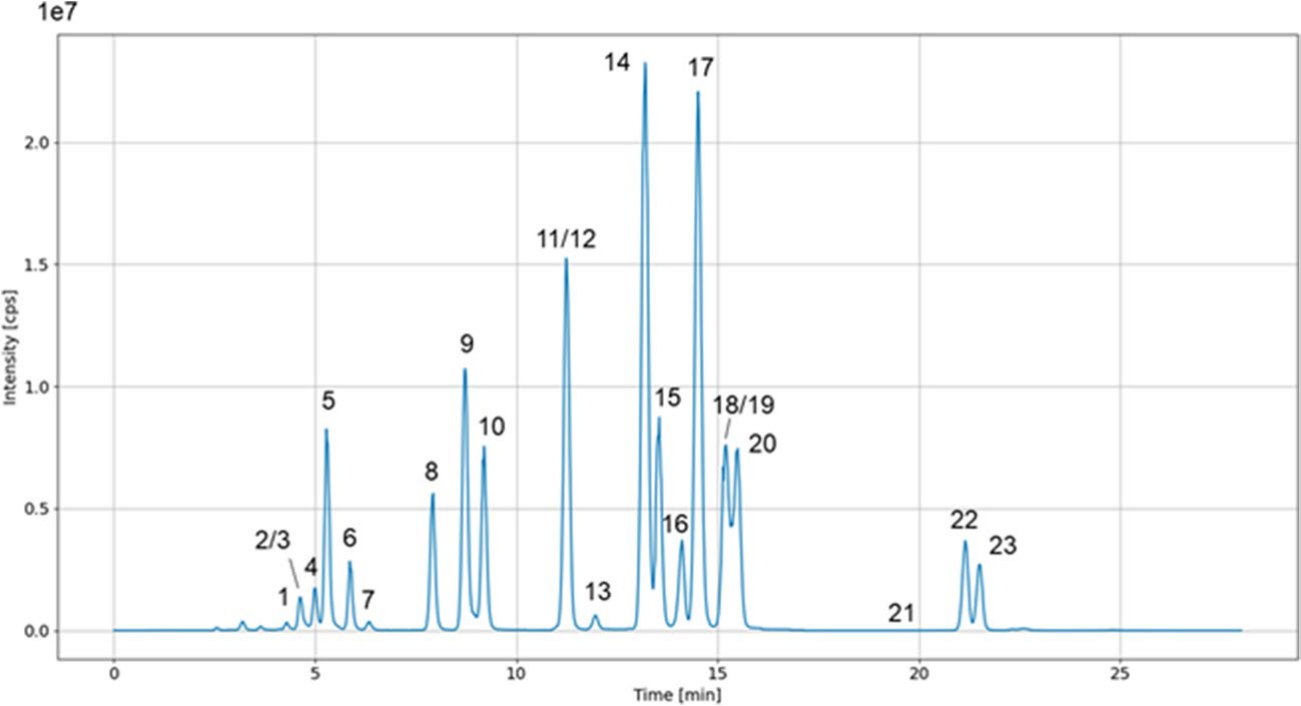
TIC of 23 analytes in a multi-component mixture for quantitative analysis using LC-APCI-MS/MS

**Fig. 2:**
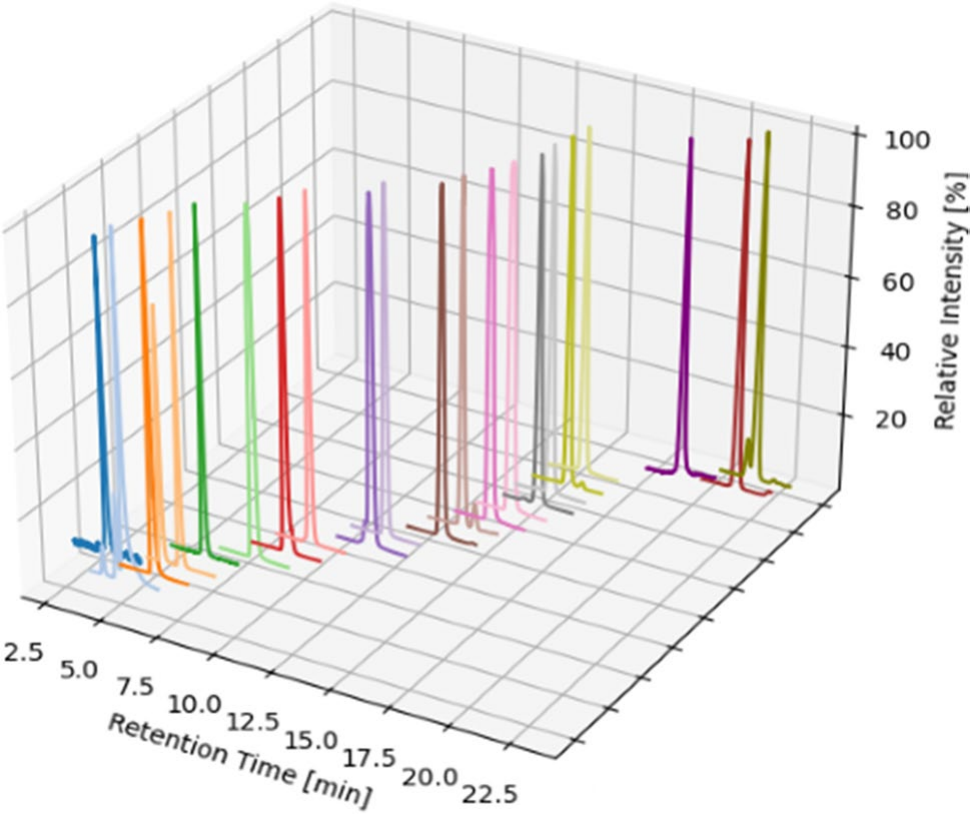
Overview of all analytes as XIC. For a better overview, the intensity is given as the relative intensity in [%]

Complete chromatographic separation was achieved for almost all 23 analytes. An oven temperature of 45 °C allowed for optimal separation of these selected analytes (Supplementary Fig. 3). The target scan time was set to 1 s and provided a sufficient number of data points for quantification. Increasing the flow rate did not significantly alter the resolution between peaks (Supplementary Fig. 4). Trans-citral **(2)** and carvacrol **(3)**, as well as CBD **(12)** and CBG **(11),** overlapped; however, they were distinguishable by mass in MRM. Figure 1 provides a chromatographic overview of the total ion chromatogram (TIC). Varying intensities were observed for all terpenes and cannabinoids, although being present at the same concentration. Two diastereomers, namely menthone **(4, 6)** and citral **(1, 3)**, were chromatographically separable. Diastereomer ratios can provide valuable information for authenticity and quality control of natural products due to stereoselective synthesis of terpenes in plants [24]. Figure 2 provides an overview of the extracted ion chromatogram (XIC) of all analytes. The chromatographic analysis included the acid forms of THC and CBD, as their total content is relevant to legal limits, and the current chemotype classification is based on their ratio. Cannabinoid acids can be detected using APCI and the same mass transitions as their neutral forms due to reproducible decarboxylation in the ionization source. Decarboxylation of the cannabinoid acids in the APCI source was further observed during compound tuning and has been described in the literature [30, 31]. In positive mode APCI ionization, cannabinoid acids undergo a partial and consistent decarboxylation, as evidenced by the lower peak areas compared to their neutral counterparts. Although APCI in negative mode offers improved ionization efficiency by generating [M-H]^+^ parent ions, the simultaneous analysis of both cannabinoids and terpenes requires a compromise [32]. Therefore, the use of neutral transitions for the cannabinoid acids in positive mode APCI allows adequate ionization and quantification of all analytes. Since the acid forms are primarily found in the plant but not in processed cannabis products such as resins and oils, the focus of this study was also on CBDV, CBN, and CBG. Nonetheless, to further prove the broad applicability and flexibility of the method, a randomly selected authentic sample was screened for CBDVA, CBNA, and CBGA using transitions from the neutral forms. All three analytes were found to be present and were baseline-separated from their neutral form (Supplementary Fig. 5). From the calibration, we were able to deduce that the areas for THCA and CBDA at the same concentration were, on average, 11 and 10 times lower than their neutral counterparts, respectively. These consistent ratios between the areas/concentrations of the acid and neutral forms could be used as a correction factor for quantification using the neutral cannabinoids as a calibration standard. However, these correction factors are dependent on ionization source and MS settings and require individual assessment. This approach would require further investigation, and could, however, be interesting from an economic point of view, as the neutral forms are more cost-effective.

### Method validation

In order to compensate for possible variations and measurement inaccuracies, we opted to select an internal standard (IS) as a correction factor. Cis-3-hexen-1-ol, propylparaben, and Δ^9^-THC-D3 were considered as potential IS candidates. In our in-house GC-FID method, cis-3-hexen-1-ol has successfully been used as an IS. However, during MRM tuning, it became apparent that very small and, therefore, unspecific fragments (< 50 Da) were selected for cis-3-hexen-1-ol. Thus, it was decided to continue with propylparaben and Δ^9^-THC-D3. Propylparaben proved suitable as an IS for cis- and trans-menthone, as well as citronellol. Δ^9^-THC-D3 served as an IS for all other analytes. In order to control for carry-over during method validation, a blank EtOH sample was injected after every five measurements. No carry-over was observed. Robustness was ensured by testing different oven temperatures and flow rates on critical pairs such as α-humulene and β-caryophyllene. Ultimately, two calibration ranges were chosen because the calibration models for the acids THCA and CBDA did not fully overlap in these ranges, and CBG showed saturation at higher concentrations. Additionally, a different weighting factor for CBD appeared better suited for both the high and low concentration ranges. Two dilution steps were necessary to cover both cannabinoids and terpenes fully. Major cannabinoids required a 100-fold dilution step as they oversaturated the detector when measured undiluted. In return, terpenes were only sufficiently detectable in undiluted samples. To compensate for this discrepancy, various injection volumes and dilutions of authentic samples were evaluated to ensure optimal coverage of all analytes. An injection volume of 5 μL provided symmetric peak shapes, and selecting two different sample preparations allowed an optimal coverage of all analytes. Unlike cannabinoids, terpenes did not ionize as efficiently, which increased their limit of detection. We further investigated whether it was possible to extend the minor calibration range to 60 μg/mL for CBD, CBDV, CBN, and Δ^9^-THC, thus eliminating the need for a second calibration. While feasible, this approach appeared impractical for our research question, as we aimed to retain the possibility of extending this method to resins or oils. In an industrial application, additional considerations would have to be made as to what type of cannabis products are to be analyzed and what the working calibration range is. Method validation took place on 3 separate days and resulted in a bias below 15% for all analytes in the minor and major calibration range. Inter- (RSD_R_) and intraday (RSD_T_) variability yielded similar results with, the exception of CBDA as a major component at the QC_low_ level, which slightly exceeded 15%. If a value did not deviate by more than 20%, which was the case for all observed analytes, it was considered acceptable. The validation results are detailed in Table 4 for the minor components and Table 5 for the major components. Further, calibration models and details can be viewed in the supplementary materials (Tables 2–3 and Figs. 1–2). Considering that terpenes are traditionally analyzed by GC, which has the advantage of high chromatographic resolution and separation performance, guaranteeing selectivity and specificity becomes even more important. Therefore, three criteria were selected to guarantee identification and quantification. To ensure analyte identity and, therefore, specificity, the relative retention time between the analyte and IS had to be within ± 0.02, both qualifier and quantifier ions had to be present, and the ion ratio had to closely match the expected ion ratio. Differences in the ion ratio < 20% were considered acceptable, < 40% marginal, and anything above 40% unacceptable. During validation all analytes met these criteria. Due to the minimal mass differences and structural similarities among terpenes, the resulting fragments alone were unreliable for identification. To exclude further interferences with other cannabinoids and their acids, a mixture consisting of 10 µg/mL THCV, THCVA, CBG, CBGA, CBDV, CBDVA, CBC, CBCA, CBNA, and Δ^8^-THC in acetonitrile was analyzed using the same method. No interferences were observed, and all cannabinoids, including their acids, were baseline separated (Supplementary Figs. 6 and 7). If required, the method presented here can be expanded to contain these 8 cannabinoids including their acids, without the need to acquire additional mass transitions. This further emphasizes the broad applicability of the method. To assess potential matrix effects such as ion enhancement and suppression, ethanolic *H. lupulus* extracts were analyzed blank and spiked at three different concentrations in the high, medium, and low range of the minor calibration, and the RE was determined. As the *H. lupulus* extract is an analyte-free matrix for cannabinoids, it was possible to observe matrix effects in great detail. The results presented in Table 4 were determined for the minor components only, as the matrix is diluted 100-fold with EtOH for the major component determination. It was therefore assumed to have a minimal effect. The RE was in an acceptable range between 80 and 120% for all analytes. Minimal to no matrix effects were observed, which is consistent with the observation, that APCI is less affected by matrix effects compared to electrospray ionization (ESI) [33].

**Table 4:** Results for method validation of terpenes and cannabinoids in the minor concentration range. Inter- (RSD_R_)- and intraday (RSD_T_) variability were used as a measurement for precision and bias for the accuracy

#	Analyte	RT [min]	Range [μg/mL]	Expected ion ratio	QC_high_			QC_med_			QC_low_			RE [%]High	RE [%] Med	RE[%]Low
					Bias [%]	RSD_R_ [%]	RSD_T_ [%]	Bias [%]	RSD_R_ [%]	RSD_T_ [%]	Bias [%]	RSD_R_ [%]	RSD_T_ [%]			
1	cis-citral	4.18	2.5–125	0.31	− 8.0	4.47	5.83	− 7.3	1.68	3.73	6.4	3.48	2.81	87.0 ± 2.9	86.2 ± 1.5	88.3 ± 4.5
2	carvacrol	4.46	5–250	0.71	− 5.4	6.65	5.87	− 3.1	9.34	8.78	8.9	4.95	8.68	99.1 ± 1.1	89.7 ± 5.3	92.1 ± 3.8
3	trans-citral	4.51	2.5–125	1.11	− 7.6	4.65	3.38	− 8.0	4.17	4.93	− 0.7	6.67	5.33	113.5 ± 4.9	113.8 ± 3.1	95.6 ± 4.0
4	trans-menthone	4.88	2.5–100	0.15	− 5.0	4.24	5.95	3.2	7.76	6.63	10.0	5.31	4.13	103.7 ± 1.1	106.7 ± 1.7	109.5 ± 5.4
5	linalool	5.17	5–250	0.03	− 5.8	3.87	4.23	0.7	4.64	7.03	10.6	6.06	4.71	86.2 ± 1.3	87.3 ± 4.3	100.2 ± 4.5
6	cis-menthone	5.74	5.5–225	0.15	− 6.4	3.44	3.79	1.6	4.83	5.25	12.3	4.73	3.96	105.4 ± 2.3	105.4 ± 2.2	104.0 ± 6.3
7	citronellol	6.17	5–250	0.67	− 7.4	2.65	3.13	− 2.7	6.32	8.56	8.3	6.64	7.89	115.9 ± 3.1	122.1 ± 2.8	106.1 ± 5.9
8	CBDV	7.77	0.1–20	0.48	1.4	2.48	4.30	2.5	2.86	4.52	2.0	8.29	6.99	85.6 ± 0.4	77.9 ± 2.4	93.5 ± 5.4
9	isobornyl acetate	8.58	5–250	0.03	− 3.4	3.83	3.77	− 3.6	4.08	4.82	4.4	7.62	7.63	95.0 ± 0.5	90.8 ± 5.0	97.9 ± 6.6
10	geranyl acetate	9.06	5–250	0.03	− 4.9	4.17	4.54	0.5	6.09	5.70	11.0	4.75	4.37	93.4 ± 1.5	91.0 ± 0.9	95.8 ± 6.1
11	CBG	11.18	0.1–20	0.40	2.8	3.03	6.01	0.5	4.27	6.07	1.0	11.80	8.87	88.5 ± 1.5	81.1 ± 2.7	89.0 ± 5.5
12	CBD	11.20	0.1–20	0.75	0.4	1.37	2.95	0.7	2.98	4.49	2.8	10.34	8.11	90.8 ± 0.4	84.8 ± 2.0	91.3 ± 5.4
13	CBDA	11.87	2–20	0.73	− 1.1	2.95	4.26	0.1	4.45	12.56	1.0	2.61	15.41	91.8 ± 1.0	81.8 ± 1.5	94.0 ± 7.3
14	sabinene	13.11	5–250	0.03	− 2.8	4.18	6.38	− 4.6	4.06	7.28	7.0	8.06	6.60	102.0 ± 3.0	98.2 ± 3.4	94.4 ± 9.4
15	myrcene	13.45	5–250	0.03	− 0.1	2.55	3.29	− 4.8	10.01	8.46	6.7	10.90	7.87	95.2 ± 3.6	91.2 ± 2.8	82.4 ± 4.6
16	CBN	14.09	0.1–20	0.72	2.5	1.70	1.79	− 1.3	2.88	2.80	0.2	9.20	6.66	97.7 ± 1.4	92.8 ± 4.9	93.8 ± 5.3
17	β-pinene	14.42	5–250	0.03	− 3.7	3.96	5.16	− 5.0	5.81	5.40	6.5	9.38	6.65	102.0 ± 4.7	96.5 ± 0.5	89.8 ± 8.4
18	limonene	15.12	5–250	0.03	− 4.8	2.08	5.33	− 0.5	4.59	10.30	4.6	4.69	4.28	108.7 ± 1.5	93.9 ± 9.8	96.3 ± 2.6
19	THC	15.30	0.1–20	0.42	2.4	0.50	2.61	1.8	3.63	4.79	3.3	9.09	7.51	98.0 ± 0.7	91.2 ± 4.2	93.6 ± 5.7
20	α-pinene	15.46	5–250	0.06	− 3.8	5.32	7.67	− 1.7	3.82	6.93	7.4	4.36	4.25	90.1 ± 2.0	85.9 ± 1.7	83.1 ± 6.1
21	THCA	19.22	2–20	0.43	− 9.9	5.36	12.00	− 9.1	8.82	12.23	− 3.6	11.23	13.57	83.1 ± 4.6	70.4 ± 8.7	84.1 ± 4.6
22	α-humulene	21.13	5–250	0.72	− 3.2	1.74	5.98	− 1.3	3.30	7.44	6.6	7.19	10.33	88.7 ± 2.1	83.7 ± 3.3	100.1 ± 3.2
23	β-caryophyllene	21.44	5–250	1.03	− 4.2	1.25	3.67	− 2.5	3.39	4.99	9.7	4.21	3.58	86.2 ± 1.2	80.4 ± 2.6	95.4 ± 4.3

**Table 5:** Results for method validation for cannabinoids in the major concentration range

#	Analyte	RT [min]	Range [μg/mL]	Expected ion ratio	QC_high_			QC_med_			QC_low_		
					Bias [%]	RSD_R_ [%]	RSD_T_[%]	Bias [%]	RSD_R_ [%]	RSD_T_[%]	Bias [%]	RSD_R_ [%]	RSD_T_[%]
8	CBDV	7.77	0.1–60	0.49	–6.5	2.77	4.43	2.9	4.74	5.14	− 11.4	5.63	6.89
11	CBG	11.18	0.1–60	0.43	–13.3	7.46	7.86	4.8	5.57	8.05	− 17.2	3.00	6.18
12	CBD	11.20	0.1–60	0.75	–0.1	2.24	3.18	9.7	3.54	4.59	− 7.8	4.83	7.04
13	CBDA	11.87	1.0–60	0.72	–5.7	5.79	7.26	–1.6	8.00	13.03	6.1	1.92	15.73
16	CBN	14.09	0.1–60	0.71	0.0	4.42	4.82	9.0	4.34	4.04	− 10.2	4.24	6.76
19	Δ^9^-THC	15.30	0.1–60	0.42	–4.6	1.41	2.72	3.2	5.13	5.14	− 12.2	3.09	5.00
22	THCA	19.22	0.1–60	0.43	–6.7	4.16	4.57	–0.3	8.28	7.76	2.8	5.38	12.99

### LC-APCI-MS/MS analysis compared to state-of-the art methods

**Fig. 3:**
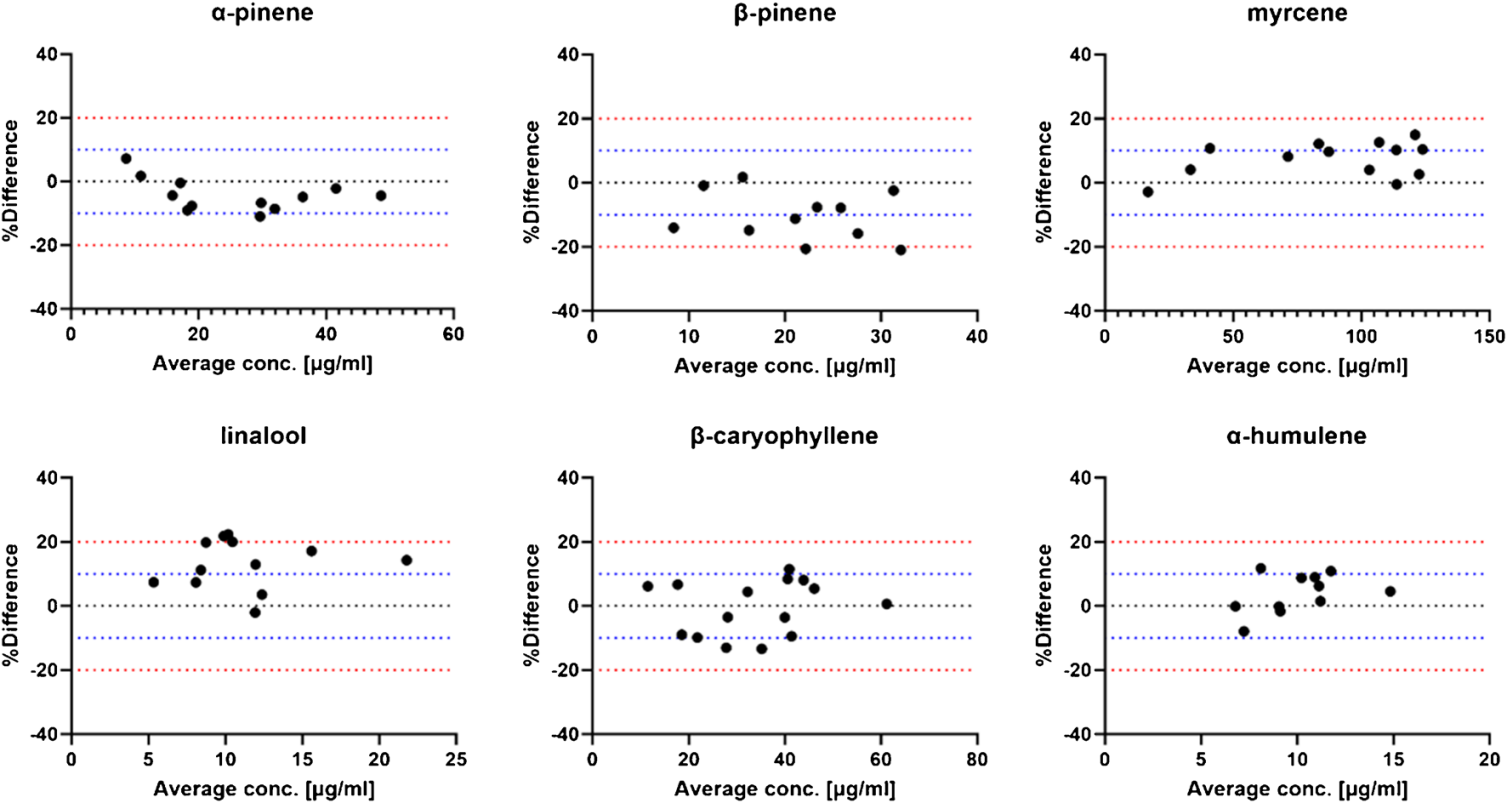
Bland–Altman plots in order to compare the concentration determination using LC-APCI-MS/MS with GC-FID of 15 authentic cannabis samples. Blue dotted lines indicate the cut-off for a 10% deviation; red dotted lines a cut-off for 20%. The average concentration [µg/ml] is depicted on the *x*-axis with the %-difference on the *y*-axis

LC is not the method of choice for the analysis of volatile compounds such as terpenes. This becomes even clearer when considering the structural similarities of terpenes and their widespread occurrence. Cannabis, in particular, is a multi-component mixture consisting of numerous cannabinoids, terpenes, and flavonoids. This special feature makes cannabis an interesting medicinal plant but also makes its analytical analysis a challenge. This multitude of chemical diverse classes often requires a combination of different analytical techniques to gain a complete understanding of the composition of cannabis, which can be both time-consuming and expensive [23, 34–36]. A single method that is able to capture as much compositional variability with minimum sample preparation in flowers of *C. sativa* would be desirable. Our study showed that terpenes can be efficiently ionized by APCI and that volatility is not a deal-breaker for the analysis with LC. Furthermore, our chromatographic method was able to separate 16 terpenes and at least 15 cannabinoids within 25 min, which is significantly shorter than most GC methods. In addition, the derivatization reactions, a process that can have side reactions and be incomplete, can be omitted. To test the reliability of the method, we compared our LC-APCI-MS/MS method with a state-of-the-art GC-FID method by randomly selecting and analyzing 15 authentic samples as a subset from the 55 authentic samples for their terpene composition. The same analytes were quantified in the samples by GC-FID, and the results of the two methods were compared using Bland–Altman plots. For reliable results, we only selected analytes that could be quantified with both methods above the LoQ in at least 10 of the samples. Samples in which the analytes were detected but were not within the calibration range were excluded from the method comparison. The calibration models, weights, and range for the analysis using GC-FID can be studied in the supplementary material Table 5. These criteria were only fulfilled by α- and β-pinene, myrcene, linalool, limonene, α-humulene, and β-caryophyllene, terpenes which are known to be major and ubiquitous components in cannabis. With the exception of limonene, all analytes were in good agreement between both methods. Figure 3 depicts the results from the method comparison using a Bland–Altman analysis. Results were considered acceptable if the percentage difference between the two methods was not greater or smaller than 20%.

Limonene concentrations determined by LC-APCI-MS/MS differed by more than double from those determined by GC-FID. However, the limonene ratio between samples appeared consistent in both the GC-FID and LC-APCI-MS/MS method. As it was initially thought that another substance was co-eluting with limonene, samples were also investigated by GC-FID. Especially α- and γ-terpinene were suspected as co-eluting analytes since both analytes are isomeric forms of limonene, sharing identical masses and fragments. However, neither were detected in any of the samples, and co-elution of these two structural isomers of limonene could be excluded. In general, the concentrations determined by LC were slightly higher than those measured by GC, which may be related to the detection method and split-injection mode. Ultimately, the co-elution of isomeric analytes is far more likely using LC than GC due to lower resolution power. For the other terpenes studied, this seemed to not be a problem. In particular, co-elutions of terpenes occurring at extremely low concentrations do not appear to have an influence on the quantification of major terpenes. This method allows faster and more reliable detection of the main constituents of *C. sativa* than traditional methods. It also has the advantage that the acid forms of the cannabinoids can be analyzed without derivatization. For the observation of extreme minor terpenes, it is still recommended to rely on the high separation power of GC. Since a minimum concentration of 0.05% v/w is required for terpenes to have a pharmacological effect, it is actually sufficient to consider and quantify only the major terpenes when cannabis is used for medicinal purposes [37]. We propose that the analysis of minor terpenes can be of particular interest when, for example, the origin or environment of the plant is of interest.

### Analysis of authentic *C. sativa* samples and chemometric analysis

**Fig. 4:**
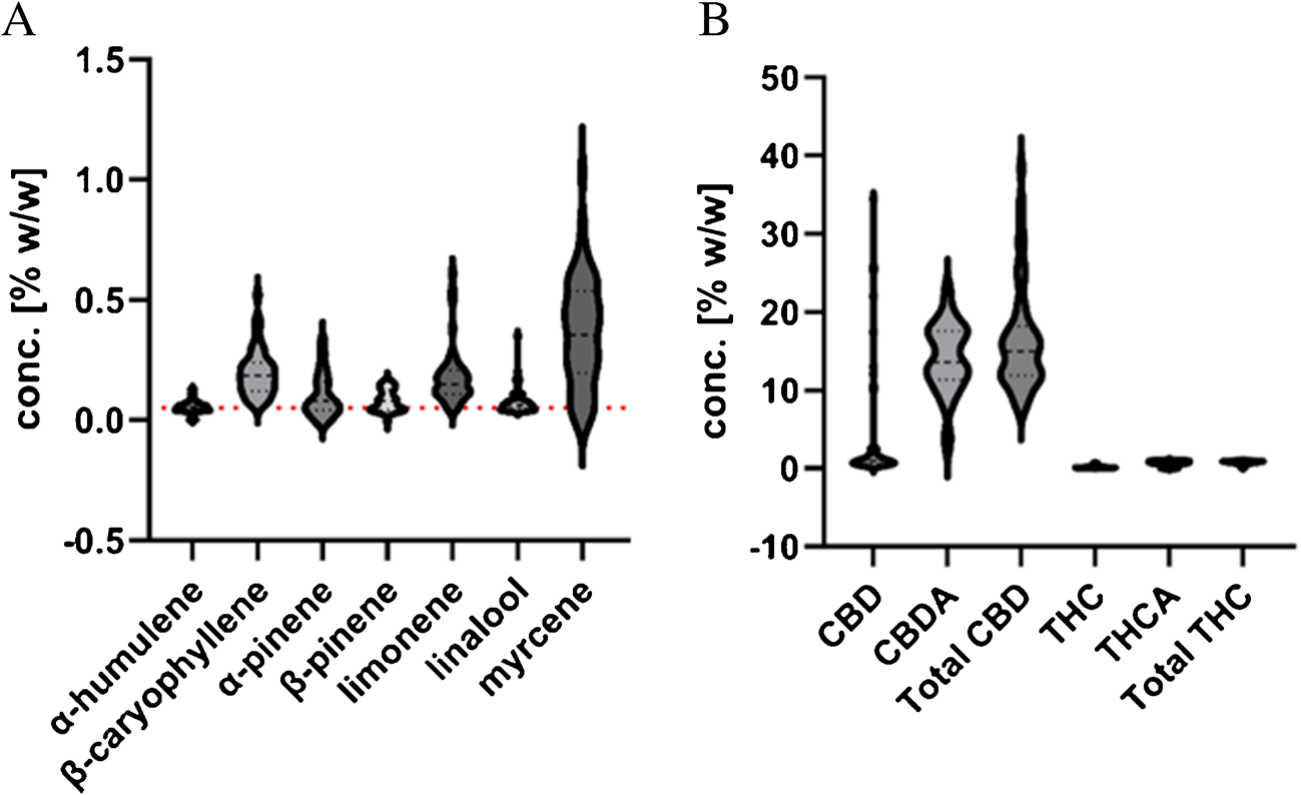
Violin plots showing the concentration of terpenes (**A**) and major cannabinoids (**B**) in % w/w of *C. sativa* flowers. The pharmacologically active threshold of 0.05% w/w for terpenes is indicated by the red dotted line [17]

**Fig. 5:**
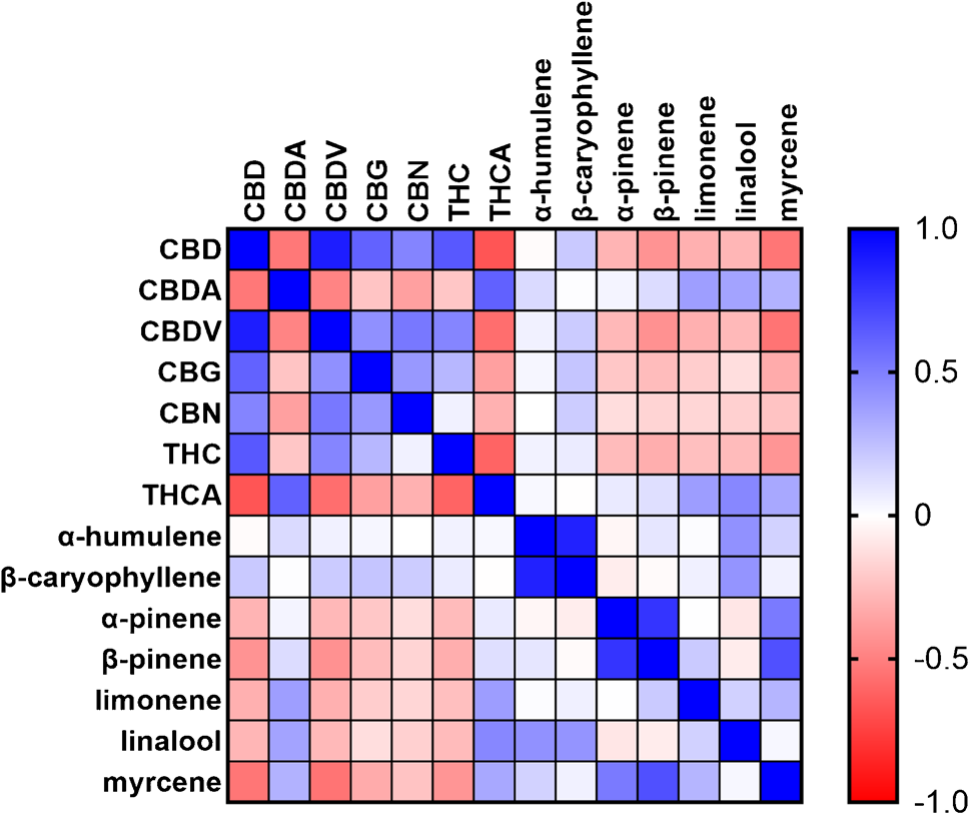
Correlation matrix after Pearson correlation of 14 quantified compounds present in 55 *C. sativa* samples. The blue coloring indicates a change in concentration in the same direction between two compounds and red an inverse correlation

**Fig. 6:**
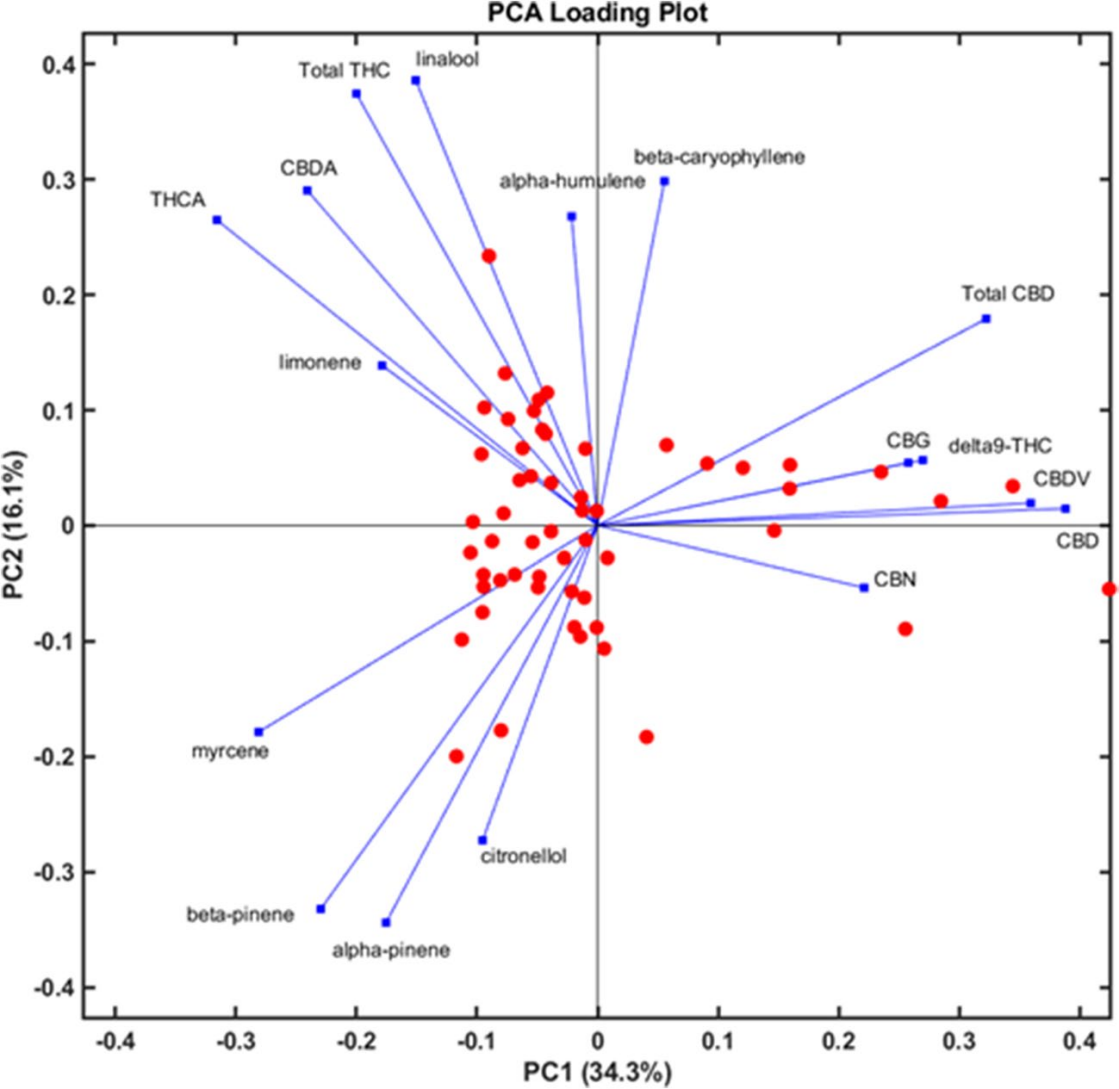
Loading plot after PCA of 55 *C. sativa* samples. Vectors in blue indicate the directionality and influence of the variables, and red dots represent individual *C. sativa* samples

A total of 55 authentic *C. sativa* flower samples were analyzed and quantified. All samples contained less than 1% total Δ^9^-THC and were therefore within the Swiss legal limit. The total CBD content varied in all samples from 7.5 to more than 30%. CBDV and CBN were detected at low levels in most samples. CBG content varied widely from trace levels to levels of around 1%. According to the Ph. Eur. 11.5 chemotype classification, all samples were assigned to the CBD-dominant type [8]. The terpene profile was dominated by myrcene, followed by β-caryophyllene, limonene, α- and β-pinene, and finally α-humulene. All other terpenes were either determined at low concentrations, below the LoQ or not detected. The complete profile of all analyzed compounds can be found in Table 5 in the Supplementary information. Figure 4A and B present the violin plots of the terpene profile and the main cannabinoid profile, respectively. Values below the LoQ were excluded from the analysis. As mentioned in the previous section, the limonene content was found to be higher than in the GC-FID measurements. As shown in Fig. 4A, limonene is one of the most important monoterpenes found in *C. sativa* flowers. This is in line with previous published reports [38]. Also, the overall terpene concentrations determined are consistent with the observation that the total terpene content is approximately 3–5% w/w of the flowers and that the CBD-dominant chemotype mostly expresses myrcene [39]. The majority of CBD and Δ^9^-THC in the extracts are in their acid forms, CBDA and THCA, respectively (Fig. 4B). This is due to the plant’s exclusive synthesis of cannabinoids in their acid forms, with decarboxylation occurring through non-enzymatic processes such as heating. Other processes, such as harvesting, storage, and processing, can also induce decarboxylation. Understanding the difference between acidic and non-acidic forms provides important information about storage and changes in cannabinoid profiles over time, which is particularly important for pharmaceutical use [10]. To get a better understanding how cannabinoid levels correlate with terpene levels, a Pearson correlation was performed. The correlation matrix is shown in Fig. 5. Interestingly, the sesquiterpenes α-humulene and β-caryophyllene increased and decreased in concentration with cannabinoid levels. In contrast, monoterpene levels demonstrated an inverse relationship with cannabinoid concentrations, with the exception of CBDA and THCA. This observation could be explained by the fact that the cannabinoid acids are decarboxylated through processes such as heating and processing, resulting in acid-free forms. However, terpenes can also evaporate during such processes, with a more significant effect on monoterpenes than sesquiterpenes. Subsequently, the terpene pattern could also serve as an indicator for the aging and processing of cannabis flowers. In addition, the double bond isomers α- and β-pinene showed the same directional concentration change as the other monoterpenes, with the exception of linalool, which showed an inverse concentration change. Since cannabinoids and terpenes originate from related building blocks (geranyl diphosphate originating from the methylerythritol 4-phosphate pathway), there may be a shortage of precursors for further terpene diversification, leading to an increased cannabinoid synthesis and decreased and selective monoterpene synthesis [40]. However, this is only speculation. A principal component analysis (PCA) was performed as an explorative analysis. The biplot, including the loading vectors, is depicted in Fig. 6. 50.4% of the total variability in the data could be explained by the first two principal components. Overall, the analysis reveals the presence of two, possibly three, distinct groups. In the two right quadrants, *C. sativa* samples appear to cluster into one group. Mostly, all samples of processed material (*C. sativa* **8**, **16**, **45**, **47**) can be found in this region. These samples are characterized by a CBD content of > 25%. It also seems to form mainly due to the contribution of minor cannabinoids such as CBG, CBDV, and CBN. Further, acid-free forms of Δ^9^-THC and CBD appear to correlate with the minor cannabinoids. The bottom left quadrant only contains vectors for monoterpenes, which are inversely correlated to the minor cannabinoids. Lastly, the top left quadrant relates THCA and CBDA with the two sesquiterpenes α-humulene and β-caryophyllene. Overall, similar conclusions can be drawn from the PCA as from the Pearson correlation. The chemical composition of the cannabis plant does not appear to occur randomly; instead depending on which analyte is up- or down-regulated, different chemical patterns emerge. Notably, correlations between the terpene and cannabinoid patterns are observed. One limitation of this study was the exclusive analysis of CBD-dominant chemotypes; however even within this chemotype, distinct subclasses exist. Given that some of the terpenes are present in pharmacologically relevant concentrations, their analysis should not be overlooked.

## Conclusion

*C. sativa* is a chemical factory for a diverse array of phytochemicals, ranging from volatile terpenes to psychoactive cannabinoids. Given the current dynamically changing legal situation, the increasing interest in *C. sativa* as a medicinal plant, and its expanding usage, a thorough understanding of its chemical composition is vital. Moreover, as a potent medicinal plant, it is central investigating the effects of storage, aging, and processing. Only through this deeper understanding can we ensure the production of high-quality phytopharmaceuticals, which is amplified by its recent inclusion into the Ph. Eur. 11.5. However, as for the pharmacopeias and legal reason, the main focus has been set on the presence of two cannabinoids. Unfortunately, this view point neglects *C. sativa*’s unique property of being a multi-component drug. The synthesis of secondary metabolites in *C. sativa* is not random—there appear to be correlations between terpenes and cannabinoids. A restructuring of the current chemotype classification has been discussed several times in the literature, and a large number of metabolic studies have suggested the inclusion of minor cannabinoids and terpenes in the classification. However, separate analyses of cannabinoids and terpenes are currently preferred. Due to the volatility and structural similarities of terpenes, successful analysis requires high-resolution separation methods. Additionally, cannabinoid acids such as CBDA and THCA are of interest, yet their analysis via GC requires prior derivatization, which can lead to side reactions and incompleteness. To our knowledge, we present the first LC-APCI-MS/MS method combining the analysis of terpenes, cannabinoids, and cannabinoid acids without the need for prior derivatization reactions. Method validation exhibited reliable results with the bias being below 15% for all analytes. Due to its selected calibration range, the presented method is suitable for analyzing both *C. sativa* flowers and processed materials. No relevant matrix effects were observed, further supporting the usage of APCI over ESI due to significantly lower susceptibility to ion enhancement and suppression. Methanol, ethanol, and water were used as solvents, making the method greener and sustainable. A drawback, however, is the inability to reliably quantify limonene due to either suspected co-elution with other terpenes or increased sensitivity of the detector. This observation is currently under further investigation. Overall, however, common terpenes and trace terpenes in *C. sativa* can be separated and quantified with results comparable to those of GC-FID. The method is rapid, with a runtime of under 30 min, and encompasses the two major classes of phytochemicals in *C. sativa*, allowing chemotype classification beyond THC and CBD using LC.

### Supplementary Information

Below is the link to the electronic supplementary material.

Supplementary file1 (DOCX 801 KB)
