# Supplementary material for: Simultaneous quantification of terpenes and cannabinoids by reversed-phase LC-APCI-MS/MS in Cannabis sativa L. samples combined with a subsequent chemometric analysis
Source: Anal Bioanal Chem. 2024 May 25;416(18):4193–206. doi: 10.1007/s00216-024-05349-y (PMC11249406; doi:10.1007/s00216-024-05349-y)
Supplement: Supplementary file 1 — Supplementary file1 (DOCX 801 KB) [file 216_2024_5349_MOESM1_ESM.docx]

Supplementary Material: Simultaneous quantification of terpenes and cannabinoids by reversed-phase LC-APCI-MS/MS in *Cannabis sativa* L. samples combined with a subsequent chemometric analysis

Justine Raeber^a^, Michael Poetzsch^b^, Anina Schmidli^a^, Sina Favrod^a^, Christian Steuer^a*^

^a^ETH Zurich, Institute of Pharmaceutical Sciences, Vladimir-Prelog-Weg 1-5/10, CH-8093 Zurich, Switzerland

^b^Swiss Drug Testing GmbH, Technoparkstrasse 2, CH-8406 Winterthur, Switzerland

^*^ Corresponding author

Tel.:+41 628385315; Fax: +41 628385399

E-mail address: christian.steuer@pharma.ethz.ch

ORCID Christian Steuer: 0000-0002-6102-3367

ORCID Justine Raeber: 0000-0003-4732-7484

ORCID Sina Favrod: 0009-0008-4435-7366


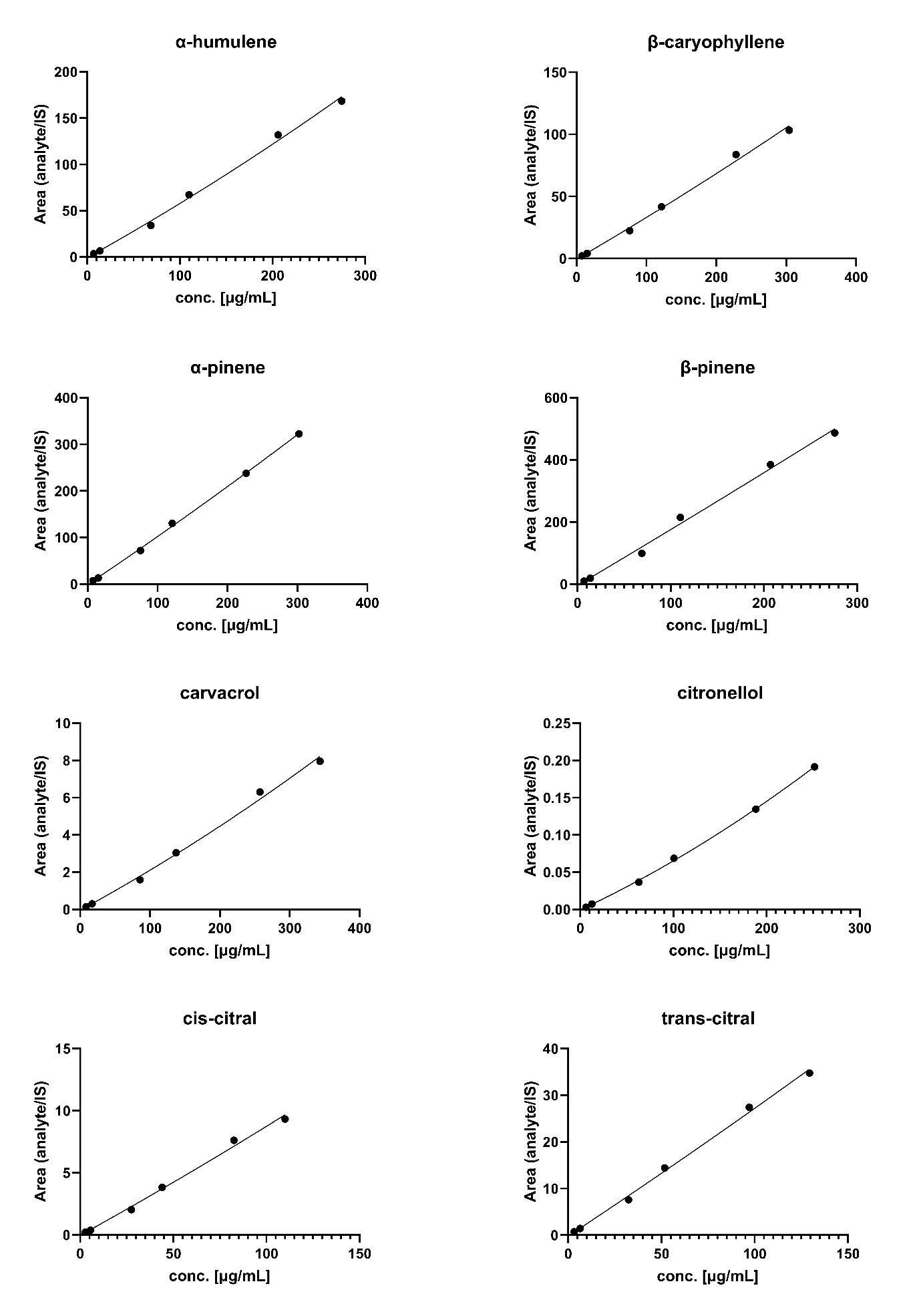

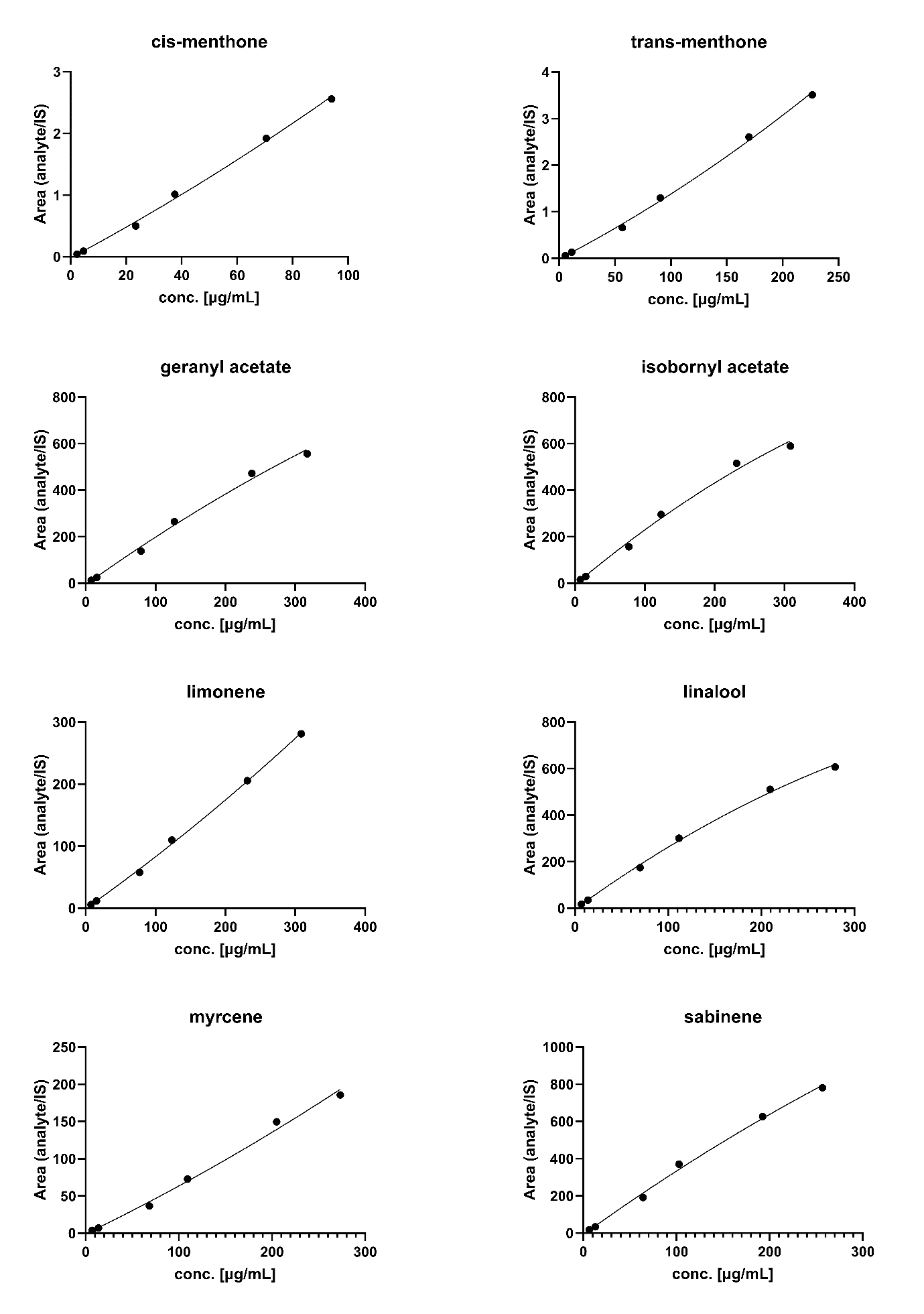

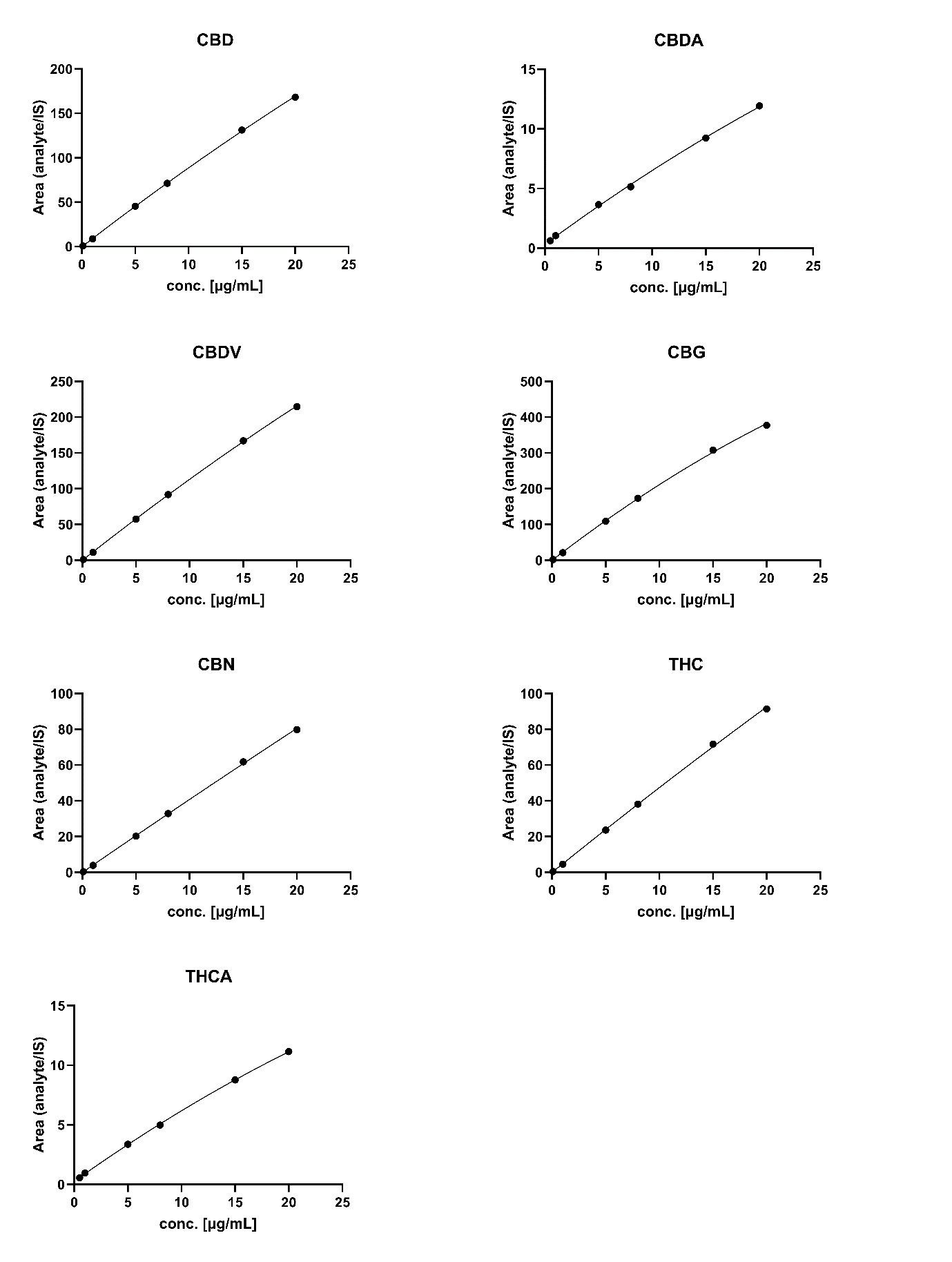


Figure 1: Calibration regression for analytes in the minor concentration range of *C. sativa*.

Table 1: Sample labels, cultivation and type.

| Sample | Cultivation | Type |
| --- | --- | --- |
| *C. Sativa 1* | Greenhouse | Flowers |
| *C. Sativa 2* | Indoor | Flowers |
| *C. Sativa 3* | Outdoor | Flowers |
| *C. Sativa 4* | unknown | Hash |
| *C. Sativa 5* | Indoor | Flowers |
| *C. Sativa 6* | Indoor | Flowers |
| *C. Sativa 7* | Greenhouse | Flowers |
| *C. Sativa 8* | unknown | Hash |
| *C. Sativa 9* | unknown | Hash |
| *C. Sativa 10* | Outdoor | Flowers |
| *C. Sativa 11* | Indoor | Flowers |
| *C. Sativa 12* | Indoor | Flowers |
| *C. Sativa 13* | Indoor | Flowers |
| *C. Sativa 14* | Outdoor | Flowers |
| *C. Sativa 15* | Outdoor | Flowers |
| *C. Sativa 16* | unknown | Hash |
| *C. Sativa 17* | Greenhouse | Flowers |
| *C. Sativa 18* | unknown | Hash |
| *C. Sativa 19* | Indoor | Flowers |
| *C. Sativa 20* | Outdoor | Flowers |
| *C. Sativa 21* | Indoor | Flowers |
| *C. Sativa 22* | Indoor | Flowers |
| *C. Sativa 23* | Outdoor | Flowers |
| *C. Sativa 24* | unknown | Hash |
| *C. Sativa 25* | unknown | Hash |
| *C. Sativa 26* | Indoor | Flowers |
| *C. Sativa 27* | Indoor | Flowers |
| *C. Sativa 28* | Greenhouse | Flowers |
| *C. Sativa 29* | unknown | Hash |
| *C. Sativa 30* | Greenhouse | Flowers |
| *C. Sativa 31* | Outdoor | Flowers |
| *C. Sativa 32* | Outdoor | Flowers |
| *C. Sativa 33* | Outdoor | Flowers |
| *C. Sativa 34* | Outdoor | Flowers |
| *C. Sativa 35* | Indoor | Flowers |
| *C. Sativa 36* | Greenhouse | Flowers |
| *C. Sativa 37* | Indoor | Flowers |
| *C. Sativa 38* | Indoor | Flowers |
| *C. Sativa 39* | Greenhouse | Flowers |
| *C. Sativa 40* | Greenhouse | Flowers |
| *C. Sativa 41* | Greenhouse | Flowers |
| *C. Sativa 42* | Outdoor | Flowers |
| *C. Sativa 43* | unknown | Hash |
| *C. Sativa 44* | Indoor | Flowers |
| *C. Sativa 45* | unknown | Hash |
| *C. Sativa 46* | Greenhouse | Flowers |
| *C. Sativa 47* | unknown | Hash |
| *C. Sativa 48* | Indoor | Flowers |
| *C. Sativa 49* | Indoor | Flowers |
| *C. Sativa 50* | Indoor | Flowers |
| *C. Sativa 51* | Outdoor | Flowers |
| *C. Sativa 52* | Indoor | Flowers |
| *C. Sativa 53* | Indoor | Flowers |
| *C. Sativa 54* | Outdoor | Flowers |
| *C. Sativa 55* | Indoor | Flowers |

Table 2: Calibration models for minor analytes in *C. sativa*.

| **Analyte** | **Calibration model** | **Weight** | **Function** | **R^2^** |
| --- | --- | --- | --- | --- |
| cis-citral | quadratic | 1/x | Y= -0.04 + 0.08*X + 0.00005*X^2 | 0.9957 |
| carvacrol | quadratic | 1/x | Y= -0.04 + 0.02*X + 0.00001*X^2 | 0.9965 |
| trans-citral | quadratic | 1/x | Y= -0.22 + 0.26*X + 0.00009*X^2 | 0.9973 |
| trans-menthone | quadratic | 1/x | Y= -0.01 + 0.01*X + 0.00001*X^2 | 0.9980 |
| linalool | quadratic | 1/x | Y= -3.71 + 2.90*X – 0.002*X^2 | 0.9984 |
| cis-menthone | quadratic | 1/x | Y= -0.02 + 0.02*X + 0.00004*X^2 | 0.9971 |
| citronellol | quadratic | 1/x | Y= -0.0006 + 0.0006*X + 0.0000007*X^2 | 0.9992 |
| CBDV | quadratic | 1/x | Y= -0.23 + 11.86*X – 0.05*X^2 | 0.9999 |
| isobornyl acetate | quadratic | 1/x | Y= -6.14 + 2.52*X – 0.0010*X^2 | 0.9947 |
| geranyl acetate | quadratic | 1/x | Y= -5.72 + 2.14*X – 0.002*X^2 | 0.9951 |
| CBG | quadratic | 1/x | Y= -0.59 + 23.22*X – 0.21*X^2 | 0.9997 |
| CBD | quadratic | 1/x | Y= -0.18 + 9.28*X – 0.04*X^2 | 0.9999 |
| CBDA | quadratic | 1/x | Y= 0.32+ 0.66*X – 0.004*X^2 | 0.9989 |
| sabinene | quadratic | 1/x | Y= -7.74 + 3.59*X – 0.002*X^2 | 0.9960 |
| myrcene | quadratic | 1/x | Y= 0.82 + 0.60*X + 0.0004*X^2 | 0.9947 |
| CBN | quadratic | 1/x | Y= -0.08 + 4.14*X -0.005*X^2 | 0.9998 |
| β-pinene | quadratic | 1/x | Y= -3.94 + 1.78*X + 0.0002*X^2 | 0.9934 |
| limonene | quadratic | 1/x | Y= -0.51 + 0.80*X + 0.0004*X^2 | 0.9986 |
| THC | quadratic | 1/x | Y= -0.09 + 4.86*X -0.01*X^2 | 0.9997 |
| α-pinene | quadratic | 1/x | Y= -0.80 + 1.01*X + 0.0002*X^2 | 0.9990 |
| THCA | quadratic | 1/x | Y= 0.26 + 0.64*X – 0.005*X^2 | 0.9995 |
| α-humulene | quadratic | 1/x | Y= -0.72 + 0.56*X + 0.0003*X^2 | 0.9964 |
| β-caryophyllene | quadratic | 1/x | Y= -0.53 + 0.33*X + 0.00008*X^2 | 0.9964 |


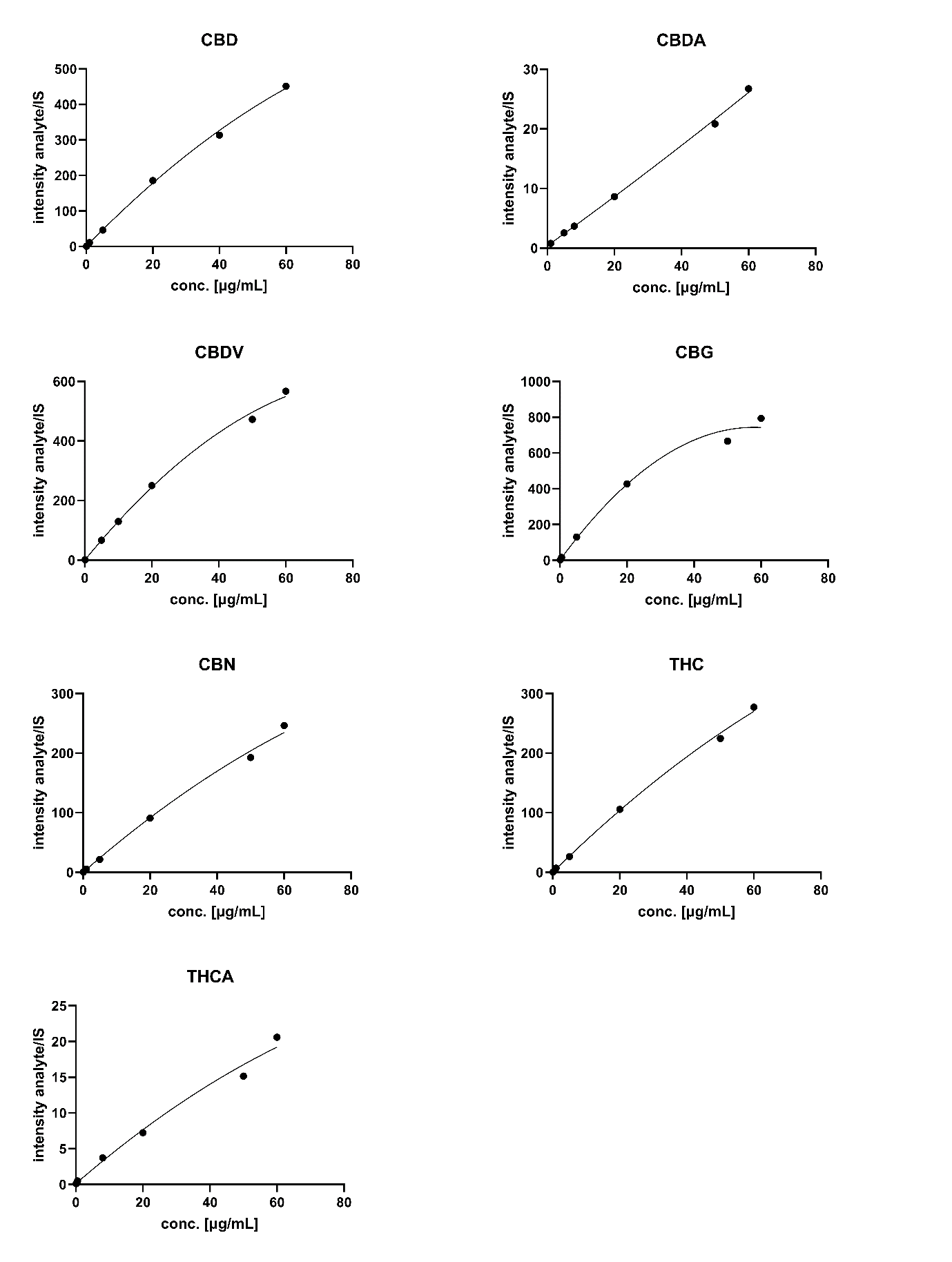


Figure 2: Calibration regression for analytes in the major concentration range of *C. sativa*.

Table 3: Calibration models for major analytes in *C. sativa*.

| **Analyte** | **Calibration model** | **Weight** | **Function** | **R^2^** |
| --- | --- | --- | --- | --- |
| CBD | Quadratic | 1/y | Y= -0.03 + 9.65*X – 0.04*X^2 | 0.9988 |
| CBDA | Quadratic | 1/x | Y= 0.40 + 0.40*X – 0.0004*X^2 | 0.9989 |
| CBDV | Quadratic | 1/x | Y= -0.14 + 13.77*X – 0.08*X^2 | 0.9988 |
| CBG | Quadratic | 1/x | Y= 0.38 + 25.62*X – 0.22*X^2 | 0.9952 |
| CBN | Quadratic | 1/x | Y= -0.10 + 4.91*X – 0.02*X^2 | 0.9934 |
| THC | Quadratic | 1/x | Y= 0.04 + 5.53*X – 0.02*X^2 | 0.9984 |
| THCA | Quadratic | 1/x | Y= 0.11 + 0.41*X – 0.001*X^2 | 0.9873 |


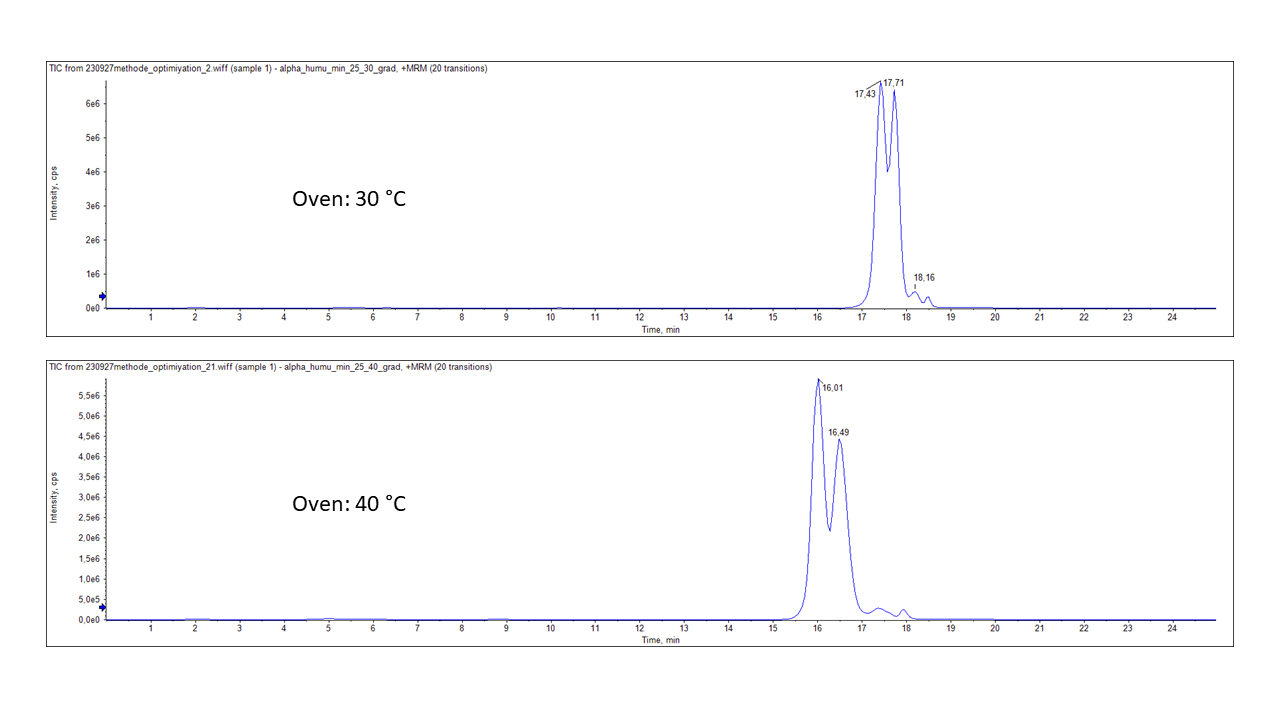


Figure 3: Effects of different oven temperatures (30 and 40 °C respectively) on the resolution of α-humulene and β-caryophyllene. A higher temperature improved separation. A final temperature of 45 °C was selected for the method due to temperature limitations of the column used.


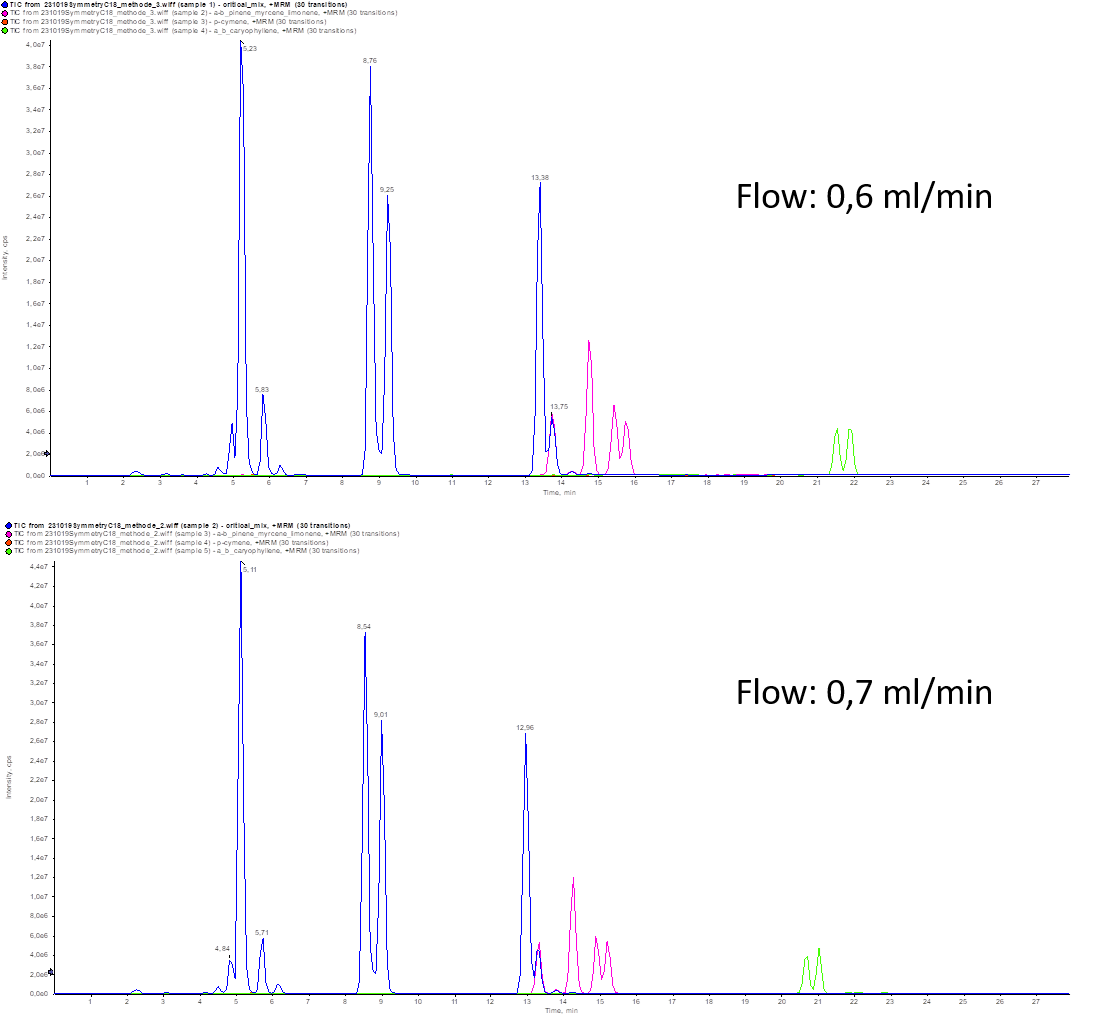


Figure 4: Different flow rates on the complete analyte mixture.

Table 4: Calibration models for terpenes cross analysed on GC-FID.

| **Analyte** | **Range [ug/mL]** | **Calibration model** | **Weight** | **R^2^** |
| --- | --- | --- | --- | --- |
| alpha-pinene | 5 - 250 | Quadratic | 1/x | 0.9971 |
| beta-pinene | 5 - 250 | Quadratic | 1/x | 0.9963 |
| sabinene | 5 - 250 | Quadratic | 1/x | 0.9850 |
| myrcene | 5 - 250 | Quadratic | 1/x | 0.9994 |
| limonene | 5 - 250 | Quadratic | 1/x | 0.9987 |
| cis-menthone | 5 – 225 | Quadratic | 1/x | 0.9986 |
| trans-menthone | 2 – 95 | Quadratic | 1/x | 0.9985 |
| linalool | 5 - 250 | Quadratic | 1/x | 0.9985 |
| isobornyl acetate | 5 - 250 | Quadratic | 1/x | 0.9983 |
| beta-caryophyllene | 5 - 250 | Quadratic | 1/x | 0.9983 |
| alpha-humulene | 5 - 250 | Quadratic | 1/x | 0.9983 |
| cis-citral | 2.5 - 110 | Quadratic | 1/x | 0.9993 |
| trans-citral | 2.5 - 110 | Quadratic | 1/x | 0.9993 |
| geranyl acetate | 5 - 250 | Quadratic | 1/x | 0.9983 |
| citronellol | 5 - 250 | Quadratic | 1/x | 0.9983 |
| carvacrol | 5 - 250 | Quadratic | 1/x | 0.9975 |

Table 5: Full quantification results expressed in % w/w of authentic *C. sativa* samples.

| Sample | CBD  [% w/w] | CBDA  [% w/w] | Total CBD  [% w/w] | CBDV  [% w/w] | CBG  [% w/w] | CBN  [% w/w] | THC  [% w/w] | THCA  [% w/w] | Total THC  [% w/w] | α-humulene  [% w/w] | β-pinene  [% w/w] | α-pinene  [% w/w] | carvacrol  [% w/w] | β-caryo-phyllene  [% w/w] | cis-citral  [% w/w] | trans-menthone  [% w/w] | Citronellol  [% w/w] | geranyl acetate  [% w/w] | isobornyl acetate  [% w/w] | limonene  [% w/w] | linalool  [% w/w] | myrcene  [% w/w] | sabinene  [% w/w] | trans-citral  [% w/w] | cis-menthone  [% w/w] |
| --- | --- | --- | --- | --- | --- | --- | --- | --- | --- | --- | --- | --- | --- | --- | --- | --- | --- | --- | --- | --- | --- | --- | --- | --- | --- |
| *C. Sativa* 1 | 0,65 | 10,48 | 9,84 | 0,001 | 0,04 | 0,0006 | 0,08 | 0,58 | 0,59 | < LoQ | 0,13 | 0,22 | n.d. | 0,08 | n.d. | n.d. | 0,0277 | n.d. | n.d. | 0,13 | 0,04 | 0,60 | n.d. | n.d. | n.d. |
| *C. Sativa* 2 | 0,64 | 17,51 | 16,00 | 0,001 | 0,18 | < LoQ | 0,08 | 0,91 | 0,88 | 0,04 | 0,15 | 0,20 | n.d. | 0,21 | n.d. | n.d. | < LoQ | n.d. | n.d. | 0,21 | 0,05 | 0,59 | n.d. | n.d. | n.d. |
| *C. Sativa* 3 | 0,95 | 12,65 | 12,04 | 0,002 | 0,07 | 0,0008 | 0,12 | 0,60 | 0,64 | 0,05 | 0,10 | 0,15 | n.d. | 0,21 | n.d. | n.d. | < LoQ | n.d. | n.d. | 0,12 | 0,05 | 0,58 | n.d. | n.d. | n.d. |
| *C. Sativa* 4 | 10,02 | 17,52 | 25,39 | 0,027 | 2,05 | 0,0716 | 0,33 | 0,43 | 0,71 | 0,03 | < LoQ | < LOQ | n.d. | 0,12 | n.d. | n.d. | n.d. | n.d. | < LoQ | 0,07 | 0,06 | n.d. | n.d. | n.d. | n.d. |
| *C. Sativa* 5 | 0,69 | 14,26 | 13,20 | 0,001 | 0,07 | 0,0009 | 0,08 | 0,81 | 0,79 | < LoQ | 0,04 | 0,08 | n.d. | 0,09 | n.d. | n.d. | < LoQ | n.d. | n.d. | 0,10 | 0,04 | 0,17 | n.d. | n.d. | n.d. |
| *C. Sativa* 6 | 0,50 | 8,73 | 8,16 | 0,001 | 0,03 | < LoQ | 0,04 | 0,58 | 0,55 | < LoQ | 0,04 | 0,05 | n.d. | 0,10 | n.d. | n.d. | n.d. | n.d. | n.d. | 0,11 | < LoQ | 0,22 | n.d. | n.d. | n.d. |
| *C. Sativa* 7 | 1,75 | 10,68 | 11,11 | 0,003 | 0,05 | 0,0023 | 0,19 | 0,52 | 0,65 | 0,03 | 0,08 | 0,09 | n.d. | 0,14 | n.d. | n.d. | n.d. | n.d. | n.d. | 0,09 | 0,05 | 0,38 | n.d. | n.d. | n.d. |
| *C. Sativa* 8 | 25,29 | 10,06 | 34,12 | 0,165 | 0,54 | 0,3627 | 0,32 | 0,13 | 0,44 | 0,06 | < LoQ | < LoQ | n.d. | 0,19 | n.d. | n.d. | n.d. | n.d. | n.d. | 0,04 | 0,06 | < LoQ | n.d. | n.d. | n.d. |
| *C. Sativa* 9 | 10,54 | 18,08 | 26,40 | 0,020 | 0,20 | 0,0265 | 0,63 | 0,39 | 0,98 | 0,05 | 0,05 | 0,08 | n.d. | 0,19 | n.d. | n.d. | n.d. | n.d. | n.d. | 0,12 | 0,04 | 0,08 | n.d. | n.d. | n.d. |
| *C. Sativa* 10 | 1,21 | 12,05 | 11,78 | 0,003 | 0,06 | 0,0007 | 0,15 | 0,60 | 0,68 | 0,06 | 0,11 | 0,14 | n.d. | 0,24 | n.d. | n.d. | n.d. | n.d. | n.d. | 0,15 | 0,06 | 0,53 | n.d. | n.d. | n.d. |
| *C. Sativa* 11 | 0,47 | 12,89 | 11,78 | 0,001 | 0,05 | 0,0005 | 0,06 | 0,79 | 0,76 | 0,04 | 0,13 | 0,18 | n.d. | 0,17 | n.d. | n.d. | 0,0341 | n.d. | n.d. | 0,18 | 0,09 | 0,65 | n.d. | n.d. | n.d. |
| *C. Sativa* 12 | 0,79 | 13,07 | 12,25 | 0,002 | 0,08 | < LoQ | 0,11 | 0,72 | 0,74 | 0,07 | 0,06 | 0,03 | n.d. | 0,30 | n.d. | n.d. | n.d. | n.d. | n.d. | 0,20 | 0,11 | 0,60 | n.d. | n.d. | n.d. |
| *C. Sativa* 13 | 0,53 | 13,60 | 12,46 | 0,001 | 0,02 | < LoQ | 0,08 | 0,78 | 0,76 | < LoQ | 0,08 | 0,08 | n.d. | 0,06 | n.d. | n.d. | < LoQ | n.d. | n.d. | 0,28 | 0,06 | 0,46 | n.d. | n.d. | n.d. |
| *C. Sativa* 14 | 2,32 | 11,38 | 12,30 | 0,003 | 0,09 | 0,0014 | 0,28 | 0,12 | 0,39 | 0,05 | 0,10 | 0,16 | n.d. | 0,17 | n.d. | n.d. | < LoQ | n.d. | n.d. | 0,09 | 0,07 | 0,59 | n.d. | n.d. | n.d. |
| *C. Sativa* 15 | 1,65 | 12,29 | 12,43 | 0,003 | 0,10 | 0,0008 | 0,22 | 0,46 | 0,62 | 0,06 | 0,12 | 0,05 | n.d. | 0,23 | n.d. | n.d. | n.d. | n.d. | n.d. | 0,61 | 0,04 | 0,45 | n.d. | n.d. | n.d. |
| *C. Sativa* 16 | > Range | 2,59 | > 30 % | 0,148 | 1,33 | 6,4436 | 0,19 | 0,01 | 0,20 | 0,04 | < LoQ | < LoQ | n.d. | 0,34 | n.d. | n.d. | n.d. | n.d. | n.d. | 0,06 | < LoQ | n.d. | n.d. | n.d. | n.d. |
| *C. Sativa* 17 | 0,89 | 9,88 | 9,55 | 0,001 | 0,03 | 0,0008 | 0,12 | 0,49 | 0,55 | 0,05 | 0,16 | 0,32 | n.d. | 0,16 | n.d. | n.d. | 0,0464 | n.d. | n.d. | 0,19 | 0,08 | 0,82 | n.d. | n.d. | n.d. |
| *C. Sativa* 18 | 17,45 | 16,46 | 31,89 | 0,103 | 0,08 | 0,2058 | 0,20 | 0,79 | 0,89 | < LoQ | < LoQ | 0,03 | n.d. | 0,10 | n.d. | n.d. | n.d. | n.d. | n.d. | 0,17 | 0,04 | 0,05 | n.d. | n.d. | n.d. |
| *C. Sativa* 19 | 0,51 | 17,99 | 16,29 | 0,002 | 0,13 | < LoQ | 0,06 | 0,95 | 0,89 | 0,07 | 0,04 | 0,04 | n.d. | 0,31 | n.d. | n.d. | n.d. | n.d. | n.d. | 0,22 | 0,20 | 0,36 | < LoQ | n.d. | n.d. |
| *C. Sativa* 20 | 1,10 | 11,87 | 11,51 | 0,002 | 0,05 | 0,0015 | 0,12 | 0,68 | 0,72 | < LoQ | < LoQ | 0,04 | n.d. | 0,12 | n.d. | n.d. | n.d. | n.d. | n.d. | 0,18 | 0,06 | 0,22 | < LoQ | n.d. | n.d. |
| *C. Sativa* 21 | 0,46 | 17,55 | 15,86 | n.d. | 0,13 | n.d. | 0,06 | 0,94 | 0,88 | n.d. | 0,04 | 0,05 | n.d. | 0,14 | n.d. | n.d. | n.d. | n.d. | n.d. | 0,21 | 0,11 | 0,34 | < LoQ | n.d. | n.d. |
| *C. Sativa* 22 | 0,58 | 17,51 | 15,94 | 0,001 | 0,14 | < LoQ | 0,07 | 1,00 | 0,94 | 0,06 | 0,04 | 0,05 | n.d. | 0,23 | n.d. | n.d. | n.d. | n.d. | n.d. | 0,26 | 0,17 | 0,56 | n.d. | n.d. | n.d. |
| *C. Sativa* 23 | 0,32 | 10,12 | 9,20 | 0,001 | 0,05 | < LoQ | 0,03 | 0,56 | 0,52 | 0,04 | 0,06 | 0,10 | n.d. | 0,17 | n.d. | n.d. | n.d. | n.d. | n.d. | 0,14 | 0,05 | 0,26 | n.d. | n.d. | n.d. |
| *C. Sativa* 24 | 13,00 | 13,27 | 24,64 | 0,022 | 0,05 | 0,1183 | 0,57 | 0,18 | 0,73 | < LoQ | < LoQ | < LoQ | n.d. | 0,08 | n.d. | n.d. | n.d. | n.d. | n.d. | 0,05 | 0,05 | < LoQ | n.d. | n.d. | n.d. |
| *C. Sativa* 25 | 12,10 | 12,81 | 23,33 | 0,062 | 0,09 | 0,1189 | 0,63 | 0,22 | 0,83 | 0,04 | < LoQ | n.d. | n.d. | 0,13 | n.d. | n.d. | n.d. | n.d. | n.d. | 0,05 | 0,06 | < LoQ | n.d. | n.d. | n.d. |
| *C. Sativa* 26 | 0,29 | 11,54 | 10,41 | 0,001 | 0,06 | < LoQ | 0,03 | 0,72 | 0,66 | 0,04 | < LoQ | < LoQ | n.d. | 0,17 | n.d. | n.d. | n.d. | n.d. | n.d. | 0,15 | 0,07 | 0,19 | n.d. | n.d. | n.d. |
| *C. Sativa* 27 | 1,34 | 11,14 | 11,11 | 0,005 | 0,07 | 0,0021 | 0,15 | 0,64 | 0,71 | n.d. | < LoQ | 0,06 | n.d. | 0,06 | n.d. | n.d. | n.d. | n.d. | n.d. | 0,10 | 0,07 | 0,11 | n.d. | n.d. | n.d. |
| *C. Sativa* 28 | 2,54 | 13,80 | 14,64 | 0,004 | 0,06 | 0,0038 | 0,28 | 0,65 | 0,85 | < LoQ | < LoQ | 0,06 | n.d. | 0,12 | n.d. | n.d. | n.d. | n.d. | n.d. | 0,17 | 0,08 | 0,27 | n.d. | n.d. | n.d. |
| *C. Sativa* 29 | 22,01 | 7,80 | 28,86 | 0,074 | 0,07 | 0,1165 | 0,67 | 0,00 | 0,67 | 0,08 | < LoQ | < LoQ | n.d. | 0,37 | n.d. | n.d. | n.d. | n.d. | n.d. | 0,08 | 0,04 | 0,11 | n.d. | n.d. | n.d. |
| *C. Sativa* 30 | 0,89 | 15,40 | 14,40 | 0,003 | 0,16 | < LoQ | 0,11 | 0,80 | 0,81 | 0,05 | 0,15 | 0,28 | n.d. | 0,26 | n.d. | n.d. | n.d. | n.d. | n.d. | 0,22 | 0,05 | 0,54 | n.d. | n.d. | n.d. |
| *C. Sativa* 31 | 2,42 | 16,09 | 16,53 | 0,005 | 0,09 | n.d. | 0,29 | 0,71 | 0,92 | < LoQ | 0,07 | n.d. | n.d. | 0,07 | n.d. | n.d. | n.d. | n.d. | n.d. | 0,38 | 0,05 | n.d. | n.d. | n.d. | n.d. |
| *C. Sativa* 32 | 1,21 | 19,91 | 18,67 | 0,002 | 0,10 | n.d. | 0,15 | 0,96 | 0,99 | 0,04 | < LoQ | n.d. | n.d. | 0,23 | n.d. | n.d. | n.d. | n.d. | n.d. | 0,21 | 0,08 | 0,50 | n.d. | n.d. | n.d. |
| *C. Sativa* 33 | 0,74 | 11,52 | 10,85 | 0,001 | 0,03 | 0,0010 | 0,07 | 0,76 | 0,74 | 0,05 | 0,05 | 0,10 | n.d. | 0,24 | n.d. | n.d. | n.d. | n.d. | n.d. | 0,12 | 0,06 | 0,33 | n.d. | n.d. | n.d. |
| *C. Sativa* 34 | 1,00 | 12,43 | 11,90 | 0,001 | 0,02 | 0,0017 | 0,10 | 0,82 | 0,82 | 0,05 | 0,16 | 0,33 | n.d. | 0,20 | n.d. | n.d. | n.d. | n.d. | < LoQ | 0,12 | 0,07 | 0,43 | n.d. | n.d. | n.d. |
| *C. Sativa* 35 | 0,73 | 22,57 | 20,53 | 0,001 | 0,15 | 0,0006 | 0,11 | 0,89 | 0,89 | < LoQ | 0,08 | 0,14 | n.d. | 0,11 | n.d. | n.d. | < LoQ | n.d. | n.d. | 0,17 | 0,08 | 0,45 | n.d. | n.d. | n.d. |
| *C. Sativa* 36 | 0,78 | 15,01 | 13,94 | 0,001 | 0,04 | < LoQ | 0,11 | 0,91 | 0,90 | 0,04 | 0,09 | 0,16 | n.d. | 0,17 | n.d. | n.d. | < LoQ | n.d. | n.d. | 0,17 | 0,06 | 0,56 | n.d. | n.d. | n.d. |
| *C. Sativa* 37 | 0,37 | 18,69 | 16,76 | 0,0005 | 0,08 | < LoQ | 0,04 | 0,95 | 0,87 | 0,04 | < LoQ | < LoQ | n.d. | 0,15 | n.d. | n.d. | n.d. | n.d. | n.d. | 0,12 | 0,11 | 0,25 | n.d. | n.d. | n.d. |
| *C. Sativa* 38 | 0,71 | 16,37 | 15,07 | 0,001 | 0,06 | < LoQ | 0,06 | 1,00 | 0,93 | < LoQ | 0,04 | < LoQ | n.d. | 0,14 | n.d. | n.d. | n.d. | n.d. | n.d. | 0,50 | 0,11 | 0,46 | n.d. | n.d. | n.d. |
| *C. Sativa* 39 | 1,19 | 15,63 | 14,90 | 0,002 | 0,05 | 0,0018 | 0,13 | 0,91 | 0,93 | 0,07 | 0,04 | 0,05 | n.d. | 0,28 | n.d. | n.d. | n.d. | n.d. | n.d. | 0,16 | 0,08 | 0,27 | n.d. | n.d. | n.d. |
| *C. Sativa* 40 | 0,98 | 21,07 | 19,46 | 0,001 | 0,08 | 0,0007 | 0,12 | 0,90 | 0,91 | 0,04 | n.d. | 0,04 | n.d. | 0,19 | n.d. | n.d. | n.d. | n.d. | n.d. | 0,54 | 0,04 | 0,43 | n.d. | n.d. | n.d. |
| *C. Sativa* 41 | 1,64 | 13,56 | 13,53 | 0,003 | 0,04 | 0,0024 | 0,19 | 0,80 | 0,90 | 0,06 | < LoQ | < LoQ | n.d. | 0,21 | n.d. | n.d. | n.d. | n.d. | n.d. | 0,11 | 0,08 | 0,24 | n.d. | n.d. | n.d. |
| *C. Sativa* 42 | 0,90 | 23,04 | 21,10 | 0,002 | 0,16 | 0,0006 | 0,10 | 0,00 | 0,10 | 0,06 | 0,13 | 0,23 | n.d. | 0,23 | n.d. | n.d. | n.d. | n.d. | n.d. | 0,22 | 0,04 | 0,46 | n.d. | n.d. | n.d. |
| *C. Sativa* 43 | 1,27 | 12,65 | 12,36 | 0,078 | 0,08 | 0,0205 | 0,15 | 0,60 | 0,67 | 0,11 | < LoQ | 0,04 | n.d. | 0,41 | n.d. | n.d. | n.d. | n.d. | n.d. | 0,12 | 0,04 | 0,06 | n.d. | n.d. | n.d. |
| *C. Sativa* 44 | 0,55 | 19,65 | 17,79 | 0,001 | 0,05 | < LoQ | 0,06 | 0,71 | 0,68 | 0,09 | 0,04 | < LoQ | n.d. | 0,30 | n.d. | n.d. | n.d. | n.d. | n.d. | 0,20 | 0,17 | 0,35 | n.d. | n.d. | n.d. |
| *C. Sativa* 45 | 34,43 | 4,46 | 38,35 | 0,077 | 1,99 | 0,0188 | 0,44 | 0,00 | 0,44 | 0,05 | < LoQ | < LoQ | n.d. | 0,41 | n.d. | n.d. | n.d. | n.d. | n.d. | 0,12 | 0,04 | 0,11 | n.d. | n.d. | n.d. |
| *C. Sativa* 46 | 2,34 | 17,94 | 18,08 | 0,008 | 0,07 | 0,0010 | 0,29 | 0,58 | 0,80 | 0,08 | 0,04 | < LoQ | n.d. | 0,28 | n.d. | n.d. | n.d. | n.d. | n.d. | 0,18 | 0,04 | 1,03 | n.d. | n.d. | n.d. |
| *C. Sativa* 47 | 25,76 | 3,94 | 29,22 | 0,118 | 0,05 | 0,2536 | 0,35 | 0,02 | 0,37 | < LoQ | < LoQ | 0,08 | n.d. | 0,12 | n.d. | n.d. | n.d. | n.d. | n.d. | 0,14 | < LoQ | 0,04 | n.d. | n.d. | n.d. |
| *C. Sativa* 48 | 0,44 | 8,07 | 7,52 | 0,001 | 0,01 | n.d. | 0,04 | 0,01 | 0,04 | < LoQ | 0,08 | n.d. | n.d. | < LoQ | n.d. | n.d. | n.d. | n.d. | n.d. | 0,08 | < LoQ | 0,31 | n.d. | n.d. | n.d. |
| *C. Sativa* 49 | 0,31 | 9,36 | 8,51 | 0,001 | 0,02 | n.d. | 0,04 | 0,55 | 0,53 | < LoQ | 0,04 | 0,09 | n.d. | 0,08 | n.d. | n.d. | < LoQ | n.d. | n.d. | 0,09 | 0,06 | 0,34 | n.d. | n.d. | n.d. |
| *C. Sativa* 50 | 0,52 | 19,74 | 17,83 | 0,001 | 0,04 | < LoQ | 0,06 | 1,02 | 0,96 | < LoQ | 0,09 | 0,19 | n.d. | 0,14 | n.d. | n.d. | < LoQ | n.d. | n.d. | 0,24 | 0,07 | 0,43 | n.d. | n.d. | n.d. |
| *C. Sativa* 51 | 1,62 | 18,11 | 17,50 | 0,003 | 0,06 | 0,0009 | 0,19 | 0,78 | 0,87 | 0,07 | 0,15 | n.d. | n.d. | 0,23 | n.d. | n.d. | 0,0354 | n.d. | n.d. | 0,18 | < LoQ | 0,66 | n.d. | n.d. | n.d. |
| *C. Sativa* 52 | 0,39 | 18,17 | 16,33 | 0,001 | 0,16 | < LoQ | 0,04 | 1,01 | 0,93 | 0,05 | 0,04 | < LoQ | n.d. | 0,18 | n.d. | n.d. | n.d. | n.d. | n.d. | 0,15 | 0,07 | 0,43 | n.d. | n.d. | n.d. |
| *C. Sativa* 53 | 0,87 | 17,93 | 16,59 | 0,002 | 0,08 | 0,0005 | 0,12 | 0,95 | 0,95 | 0,06 | < LoQ | n.d. | n.d. | 0,20 | n.d. | n.d. | n.d. | n.d. | n.d. | 0,13 | 0,14 | 0,24 | n.d. | n.d. | n.d. |
| *C. Sativa* 54 | 0,98 | 16,77 | 15,68 | 0,003 | 0,03 | < LoQ | 0,13 | 0,73 | 0,77 | 0,06 | 0,14 | 0,27 | n.d. | 0,25 | n.d. | n.d. | < LoQ | n.d. | n.d. | 0,18 | 0,04 | 0,72 | n.d. | n.d. | n.d. |
| *C. Sativa* 55 | 0,45 | 16,28 | 14,73 | 0,001 | 0,03 | 0,0005 | 0,05 | 0,95 | 0,88 | 0,13 | 0,06 | 0,04 | n.d. | 0,52 | n.d. | n.d. | n.d. | n.d. | n.d. | 0,25 | 0,35 | 0,14 | n.d. | n.d. | n.d. |


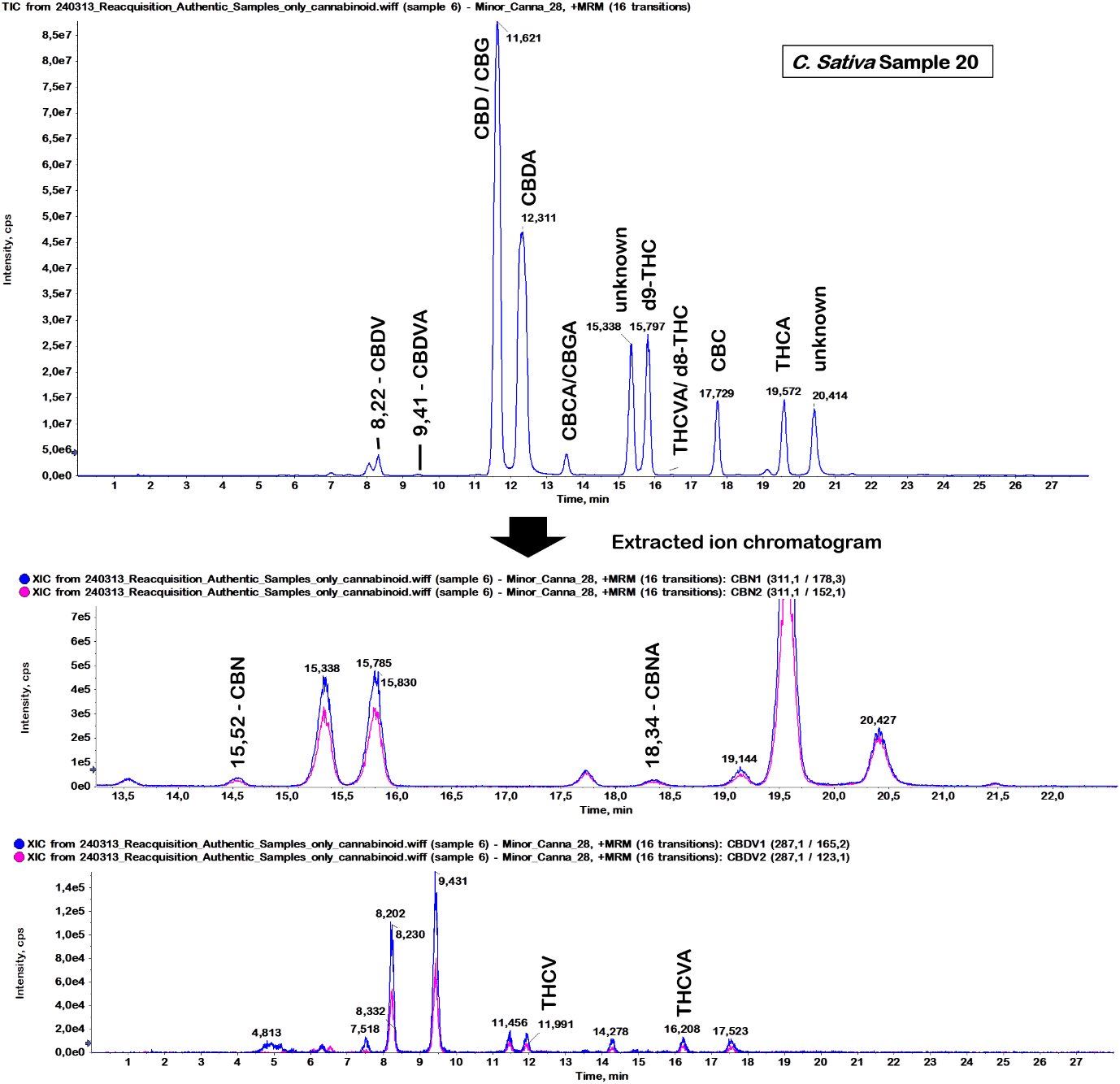


Figure 5: TIC and XIC of sample *C. sativa* 20.


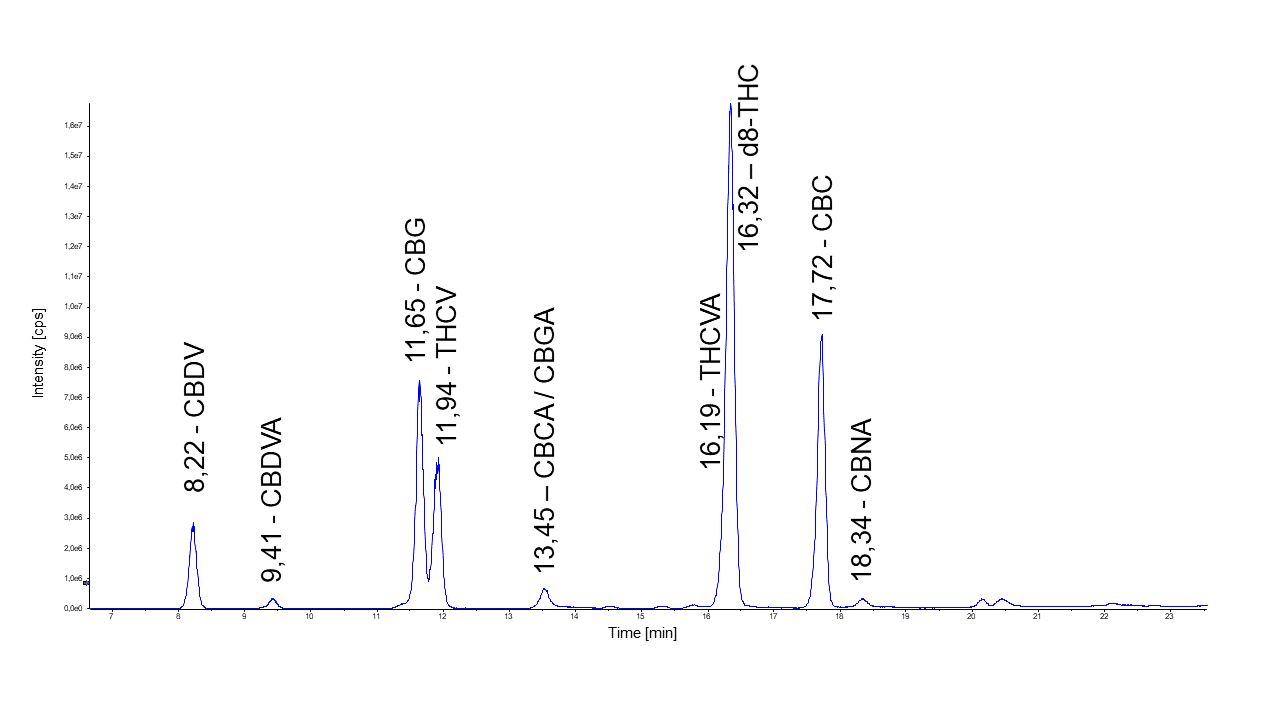


Figure 6: TIC of analyte mixture in order to study possible interferences between cannabinoids.


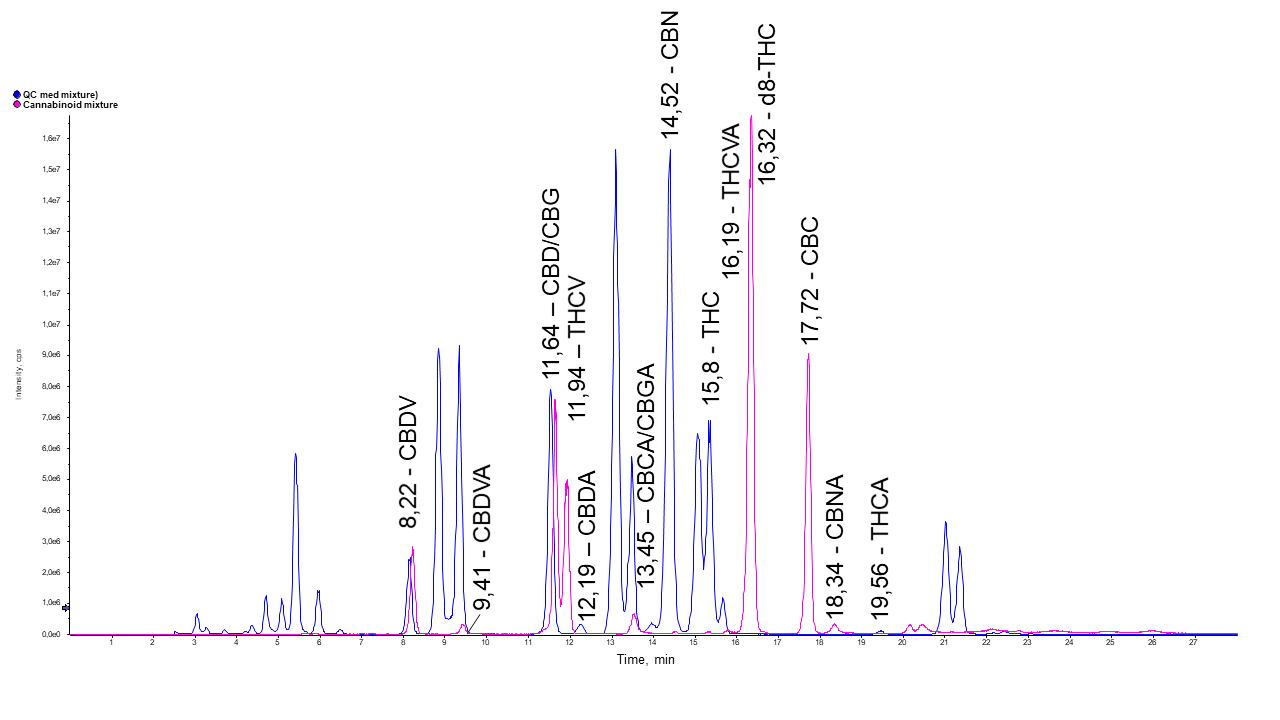


Figure 7: Overlay of the TIC of the QC med mixture as well as the cannabinoid mixture. All analytes can either be distinguished via mass transitions or are baseline separated.
